# Proteogenomic Characterization of High-Grade Lung Neuroendocrine Carcinoma Deciphers Molecular Diversity and Potential Biomarkers of Different Histological Subtypes in Chinese Population

**DOI:** 10.34133/research.0671

**Published:** 2025-04-14

**Authors:** Zicheng Zhang, Xi Wu, Siqi Bao, Xujie Sun, Fan Yang, Yibo Zhang, Zijian Yang, Liujin Zhang, Ruanqi Chen, Puyuan Xing, Junling Li, Meng Zhou, Lin Yang

**Affiliations:** ^1^ School of Biomedical Engineering, Wenzhou Medical University, Wenzhou 325027, P. R. China.; ^2^ Department of Comprehensive Oncology, National Cancer Center/National Clinical Research Center for Cancer/Cancer Hospital, Chinese Academy of Medical Sciences and Peking Union Medical College, Beijing 100021, P. R. China.; ^3^ Department of Pathology, National Cancer Center/National Clinical Research Center for Cancer/Cancer Hospital, Chinese Academy of Medical Sciences and Peking Union Medical College, Beijing 100021, P. R. China.; ^4^ Department of Oncology, National Cancer Center/National Clinical Research Center for Cancer/Cancer Hospital, Chinese Academy of Medical Sciences and Peking Union Medical College, Beijing 100021, P. R. China.

## Abstract

High-grade lung neuroendocrine carcinomas (Lu-NECs) are clinically refractory malignancies with poor prognosis and limited therapeutic advances. The biological and molecular features underlying the histological heterogeneity of Lu-NECs are not fully understood. In this study, we present a multi-omics integration of whole-exome sequencing and deep proteomic profiling in 93 Chinese Lu-NECs to establish the first comprehensive proteogenomic atlas of this disease spectrum. Our analyses revealed a high degree of mutational concordance among the subtypes at the genomic level; however, distinct proteomic profiles enabled a clear differentiation of histological subtypes, unveiling subtype-specific molecular and biological features related to tumor metabolism, immunity, and proliferation. Furthermore, *RB1* mutations confer divergent prognostic effects through subtype-specific *cis-* and *trans-*proteomic regulation. In addition, we identified potential protein biomarkers for histological subtype classification and risk stratification, which were validated by immunohistochemistry in an independent cohort. This study provides a valuable proteogenomic resource and insight into Lu-NEC heterogeneity.

## Introduction

Neuroendocrine neoplasms (NENs) are a heterogeneous group of tumors arising from neuroendocrine cells distributed throughout the body and can be classified into different types according to their origin [1,2]. Lung neuroendocrine neoplasms (Lu-NENs) constitute approximately 20% of all NEN cases and are categorized into low-grade typical carcinoid (TC), intermediate-grade atypical carcinoid (AC), and high-grade neuroendocrine carcinoma (NEC) according to the latest World Health Organization (WHO) classification of thoracic tumors (5th edition, 2021) [3]. High-grade lung neuroendocrine carcinomas (Lu-NECs), characterized by rapid progression and poor prognosis, represent a particularly aggressive subset of lung NENs and are composed of small cell lung carcinoma (SCLC) and large cell neuroendocrine carcinoma (LCNEC) [4,5]. Despite advances in diagnostic and therapeutic strategies, Lu-NECs remain challenging due to their complex molecular and histological subtype heterogeneity [6–9].

While LCNEC and SCLC share some pathological features, including neuroendocrine differentiation and high proliferation rates, they differ markedly in cellular morphology and chemotherapeutic response [10–14], reflecting their distinct biological or molecular characteristics. Despite this, they are both classified under the umbrella of high-grade NENs, highlighting the complexity and variability within Lu-NENs. Recent molecular studies have begun to unravel the genomic and transcriptomic molecular characteristics of these histological types, identifying 2 distinct molecular subtypes of LCNEC and 4 of SCLC [6,9]. For example, type I LCNECs share genomic alterations with lung adenocarcinomas and squamous cell carcinomas, as well as a neuroendocrine profile with SCLC tumors. In contrast, type II LCNECs are genetically similar to SCLC but are distinctly different, with reduced expression of neuroendocrine markers and increased activity of the NOTCH signaling pathway [9]. In addition, the co-occurrence of both SCLC and LCNEC components (combined SCLC and LCNEC, cSCLC-LCNEC) within the same tumors further complicates classification efforts and highlights the blurred molecular boundaries between these entities [15]. While recent studies have provided initial insights into the genomic and transcriptomic landscape of Lu-NECs, they fall short of capturing the full range of functional effects and downstream biological processes, as the ultimate mediators of the phenotype, proteins, remain underexplored. Recent advances in the field of proteogenomics, combining proteomic and genomic analyses, have begun to unravel the complex molecular and functional landscapes of various cancer types, providing new insights into their biology, diagnosis, and treatment. However, the proteogenomic landscape of Lu-NECs, particularly in SCLC, LCNEC, and their mixed subtypes, especially in Chinese patients, has not been thoroughly investigated.

In this study, we performed an integrated genomic and proteomic landscape analysis of 93 Chinese Lu-NEC specimens with long-term follow-up. The comparative proteogenomic profile across different histological types not only delineates shared and unique genomic and proteomic molecular alterations, as well as aberrations in signaling pathways, but also unravels the impact of their complex interplay on tumor phenotype diversity and clinical outcomes. Ultimately, our study provides a valuable resource and new functional insights into Lu-NEC biology, paving the way for more personalized and effective therapeutic strategies for patients with Lu-NEC.

## Results

### Clinical and molecular features of Chinese Lu-NECs

To delineate the comprehensive proteogenomic landscape of Lu-NECs in the Chinese population, we performed whole-exome sequencing (WES) and proteomic profiling of formalin-fixed paraffin-embedded (FFPE) tumor samples obtained from 93 Lu-NECs diagnosed at the Cancer Hospital, National Cancer Center of China, Chinese Academy of Medical Sciences, including 42 LCNECs, 30 SCLCs, and 21 cSCLC-LCNECs (Fig. S1A). The clinicopathological characteristics of all patients were summarized in Table S1. Across the 3 histological types of Lu-NECs, the median age was 62 years, ranging from 48 to 75 years for LCNEC, 53 to 77 years for cSCLC-LCNEC, and 40 to 77 years for SCLC. Smoking prevalence was highest in cSCLC-LCNEC patients (100%), followed by LCNEC (95.2%) and SCLC (63.3%). Postoperative adjuvant therapy was administered to 75.3% of the Lu-NEC cohort.

A total of 93 WES and 76 proteomic samples were included for proteogenomic analysis after stringent quality control. To address the potential challenges of low-quality FFPE samples, pathologists used microscopic counting to determine tumor purity, ensuring a minimum tumor purity threshold of 60% in all FFPE samples (Fig. S1B). Using the protocol described in the Methods section, we successfully identified a total of 33,986 high-quality somatic variants, comprising 13,998, 8,333, and 11,655 mutations with median values of 319, 350, and 351.5 in LCNEC, cSCLC-LCNEC, and SCLC, respectively (Figs. S1C and S2A and B). These mutations included missense mutations, nonsense mutations, splice site mutations, frameshift deletions, in-frame deletions, and insertions, as well as multi-hit and translation start site mutations. A total of 81,120 peptides corresponding to 9,707 proteins were identified in the proteome analysis. According to the filtering criteria described in the Methods section, 6,979 proteins were retained for further downstream analysis (Fig. S1D). Notably, all peptides exceeded 7 amino acids in length, and no substantial batch effects were observed in the protein intensity distribution among Lu-NECs (Figs. S1E and S3A to C). Analysis of associations between samples within identical histological subtypes showed a high reproducibility of the proteomic data, as evidenced by median Spearman correlation coefficients of *r* = 0.871, 0.888, and 0.906. However, *t*-distributed stochastic neighbor embedding (*t*-SNE) analysis of quantified proteins across all samples revealed a clear distinction in the proteomic profiles of different histological subtypes of Lu-NECs (Fig. S4A to D).

### A spectrum of diverse genomic alterations in Lu-NECs

**Fig. 1.**
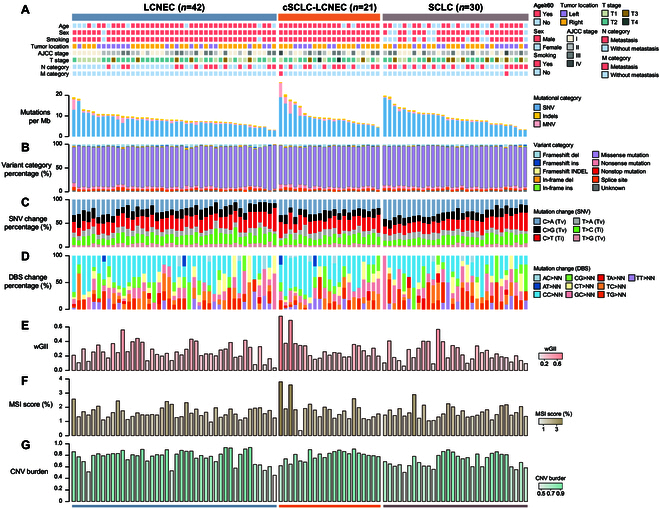
The heterogeneity of genomic alterations among Lu-NECs. (A) Bar plots showing the number of genomic mutations per megabase (TMB) in each Lu-NEC sample based on WES data. (B) Stacked bar plots showing the percentage of variant categories in each Lu-NEC sample. (C) Percentage of the 6 pyrimidine point mutation (SNV) categories in each Lu-NEC sample. (D) Percentage of the doublet base substitution (DBS) in each Lu-NEC sample. (E) Bar plot showing the distribution of wGII between LCNEC, cSCLC-LCNEC, and SCLC. (F) Bar plot showing the distribution of MSI score between LCNEC, cSCLC-LCNEC, and SCLC. (G) Bar plot showing the distribution of CNV burden between LCNEC, cSCLC-LCNEC, and SCLC.

The comprehensive mutational landscape of Lu-NECs revealed distinct genomic characteristics among LCNEC, SCLC, and cSCLC-LCNEC (Fig. 1). We observed differences in tumor mutational burden (TMB), with cSCLC-LCNEC having the highest TMB (median of 9.024) compared to LCNEC (8.131) and SCLC (8.821) (Fig. 1A and Fig. S5A). Furthermore, cSCLC-LCNEC had a higher number of single-nucleotide variants (SNVs) (median of 334) compared to LCNEC (297.5) and SCLC (328). However, LCNEC showed a higher frequency of insertion–deletion mutations (InDels: 17.5) and multiple-nucleotide variants (MNVs: 25) (Fig. S5A). As shown in Fig. 1B, a large proportion of somatic mutations for all Lu-NECs were identified as missense mutations (LCNEC: 83.5%, cSCLC-LCNEC: 82.9%, and SCLC: 84.5%), followed by nonsense mutations (LCNEC: 5.2%, cSCLC-LCNEC: 6.0%, and SCLC: 5.3%).

The distribution of transitions (Ti: C>T and T>C) and transversions (Tv: C>A, C>G, T>A, and T>G) varied among Lu-NECs (Fig. 1C). Notably, the frequency of C>A (Tv) mutations was highest in SCLC with a median value of 88.5 (Fig. S5B). Additionally, the median Ti/Tv ratio in LCNEC was 0.843, which was higher compared to both cSCLC-LCNEC (0.781) and SCLC (0.670) (Fig. S5C). Interestingly, doublet base substitutions showed a lower frequency in all Lu-NEC samples, with no statistically significant differences observed between LCNEC, cSCLC-LCNEC, and SCLC (Fig. 1D and Fig. S5D).

We also performed a comparative analysis of genomic stability measures, including weighted genome instability index (wGII), microsatellite instability score (MSI score), and copy number variation (CNV) burden, among LCNEC, cSCLC-LCNEC, and SCLC (Table S2 and Fig. 1E to G). The results showed that both wGII (medians of 0.250 for LCNEC, 0.258 for cSCLC-LCNEC, and 0.195 for SCLC) and MSI score (medians of 1.565 for LCNEC, 1.800 for cSCLC-LCNEC, and 1.510 for SCLC) were relatively consistent across Lu-NEC subtypes (Fig. S5E and F), with statistically significant differences in CNV burden. Specifically, SCLC had a median CNV burden of 0.684, which was significantly lower than both LCNEC (median, 0.801) and cSCLC-LCNEC (median, 0.815) (Kruskal–Wallis test, *P* = 0.014; Fig. S5G). To determine how the significantly different mutations (MNV frequency and CNV burden) specifically affected the pathways in Lu-NECs, we performed the enrichment analysis in 17 highly correlated proteins. As shown in Fig. S5H, both MNV frequency and CNV burden correlated proteins were enriched in protein degradation, nucleotide metabolism, and protein synthesis-related processes (*FDR* < 0.05).

### Differential effects of RB1 mutation in different histological types of Lu-NECs

**Fig. 2.**
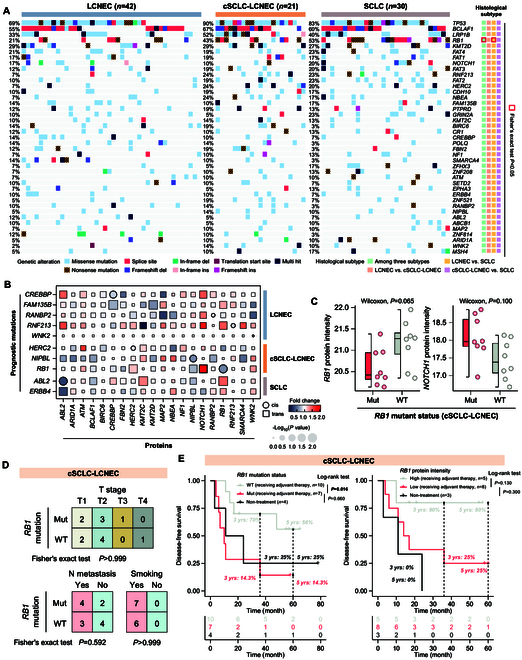
RB1 mutation performed different effects between LCNEC, cSCLC-LCNEC, and SCLC. (A) Oncoplot showing the frequency and types of somatic mutations of tumor driver genes among Lu-NECs. The different mutation distribution of driver genes was calculated using Fisher's exact test between LCNEC, cSCLC-LCNEC, and SCLC (*P* < 0.05). (B) The *cis-* and *trans-*effects of significant prognostic driver genes (*y* axis) at the proteomic level (*x* axis). The *P* values were calculated with the Wilcoxon test and transferred as −log_10_(*P* values). Fold changes were calculated as the ratio of protein intensity in mutant versus WT. (C) Box plots showing the differential protein intensity of *RB1* and *NOTCH1* between *RB1* mutant and WT in cSCLC-LCNEC. (D) Distribution of *RB1* mutant status among clinicopathological features with Fisher's exact test. (E) The Kaplan–Meier curves showing the different prognosis between *RB1* subtypes based on *RB1* mutation and *RB1* protein intensity. *P* values were calculated using the log-rank test.

To characterize the alterations in genetic information within Lu-NECs, we statistically analyzed the mutation frequency by examining the overlap between known cancer driver genes and Lu-NECs hotspot genes in each histological subtype. The results, as shown in Fig. 2A, revealed high-frequency mutations in *TP53* (LCNEC: 69%, cSCLC-LCNEC: 90%, and SCLC: 83%), *LRP1B* (LCNEC: 33%, cSCLC-LCNEC: 52%, and SCLC: 40%), *RB1* (LCNEC: 21%, cSCLC-LCNEC: 43%, and SCLC: 53%), *KMT2D* (LCNEC: 29%, cSCLC-LCNEC: 29%, and SCLC: 27%), and *NOTCH1* (LCNEC: 17%, cSCLC-LCNEC: 10%, and SCLC: 20%), consistent with previous studies [16–18]. Univariate Cox regression analyses were performed for each gene mutation in the 3 histological subtypes to examine the association between cancer driver gene mutations and prognosis. LCNEC had a significantly lower frequency of *RB1* mutations compared to both cSCLC-LCNEC and SCLC (Fisher's exact test, *P* < 0.05), and *RB1* mutations were identified as an unfavorable prognostic marker in cSCLC-LCNEC (Fig. S6A). Notably, 9 additional cancer driver genes (*ABL2*, *CREBBP*, *ERBB4*, *FAM135B*, *HERC2*, *NIPBL*, *RANBP2*, *RNF213*, and *WNK2*) were associated with unfavorable prognosis in LCNEC, cSCLC-LCNEC, and SCLC, respectively (Fig. S6A). Furthermore, we found that patients with comutation of *TP53* and *RB1* in cSCLC-LCNEC and SCLC had a worse DFS prognosis than LCNEC patients (3-year DFS rates: 75% in LCNEC, 16.7% in cSCLC-LCNEC, and 50% in SCLC; Fig. S6B).

Next, we comprehensively investigated the cis- and *trans*-effects between prognostic cancer driver genetic alterations and protein intensity. Compared to *cis*-effects, we observed more significant changes in protein intensity for *trans*-effects (Fig. 2B). For example, the *trans*-effect analysis revealed higher NOTCH1 intensity associated with *RB1* mutations, suggesting a potential impact of *RB1* in regulating NOTCH1 protein intensity in cSCLC-LCNEC (Fig. 2C). Notably, only one *cis-*effect was marginally significant in Lu-NECs: *RB1* (cSCLC-LCNEC) (Fig. 2C and Fig. S6C). Importantly, *RB1* mutation status was statistically independent of clinical variables such as T stage, N metastasis, and smoking history in cSCLC-LCNEC patients (Fig. 2D). These findings suggest that *RB1* mutations contribute to decreased RB1 protein levels and increased NOTCH1 protein levels. Given the association of *RB1* mutations and decreased RB1 protein intensity with unfavorable outcomes in cSCLC-LCNEC [3-year DFS rate: 70% in *RB1* wild type (WT) versus 14.3% in *RB1* mutant, 80% in RB1 high versus 25% in RB1 low, and 5-year DFS rate: 56% in *RB1* WT versus 14.3% in *RB1* mutant, 80% in RB1 high versus 25% in RB1 low], while the prognosis of receiving adjuvant therapy in samples with *RB1* mutation or decreased RB1 intensity was similar to that of untreated samples (log-rank *P* > 0.1; Fig. 2E). In contrast, the decreased RB1 expression of SCLC patients was associated with an unfavorable prognosis, which was confirmed in the George cohort (log-rank *P* = 0.038) and the Jiang cohort (log-rank *P* = 0.008; Fig. S6D).

### Proteomic characterization of Chinese Lu-NECs

**Fig. 3.**
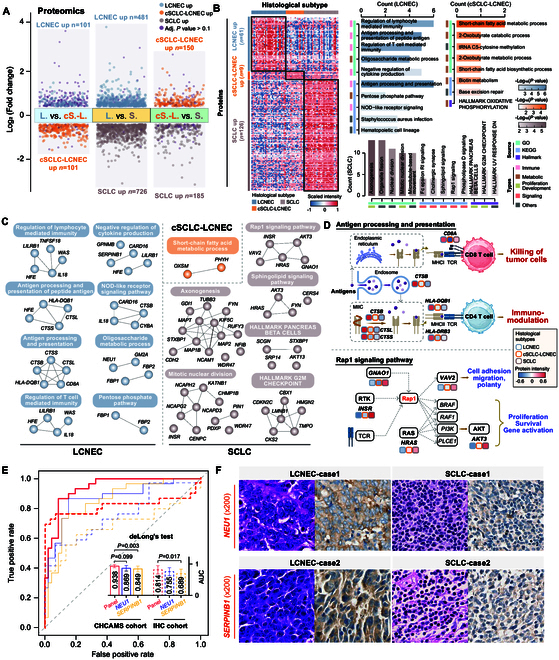
Proteomic landscape and characteristics of 3 Lu-NEC subtypes. (A) Differential protein intensity analysis showing up- and down-regulated proteins among 3 Lu-NEC subtypes. *P* values were calculated with the Wilcoxon test and adjusted by the FDR method. The adjusted *P* values > 0.1 are shown in purple, indicating nonsignificance. (B) Heatmap showing the intensity of specific up-regulated proteins in Lu-NECs (left panel). Bar plots showing the enrichment of biological features including GO BP, KEGG pathway, and Hallmark gene sets in each Lu-NEC subtype (*FDR* < 0.1). (C) Protein–protein interaction analysis of enriched protein sets (B) in 3 Lu-NEC subtypes. (D) Schematic representation of the effect of key pathways in LCNEC versus cSCLC-LCNEC versus SCLC on T cells and tumor proliferation. (E) ROC analyses for differentiating patients with LCNEC from patients with SCLC in the CHCAMS cohort (solid lines) and the independent IHC validation cohort (dashed lines) using the 2-protein panel. (F) Representative hematoxylin and eosin (H&E) and IHC staining images of protein markers (*NEU1* and *SERPINB1*) in LCNEC and SCLC (200× magnification).

The proteomic analysis quantified a total of 6,979 proteins in Lu-NECs. Unsupervised clustering revealed distinct proteomic profiles for each histological subtype, indicating their unique biological characteristics (Fig. S7A). Differential intensity analyses were performed between histological subtypes to identify proteins specifically up-regulated in each subtype (Fig. 3A). Specifically, we identified 61, 9, and 126 histology-specific up-regulated proteins for LCNEC, cSCLC-LCNEC, and SCLC, respectively (Fig. S7B). Functional enrichment analyses of these proteins revealed that LCNEC-specific proteins were predominantly associated with immune and metabolic functions, including regulation of lymphocyte-mediated immunity, antigen processing and presentation, regulation of T cell-mediated immunity, oligosaccharide metabolism, and pentose phosphate pathway (PPP) (Fig. 3B and Fig. S7C). In contrast, SCLC-specific proteins revealed significant enrichment in signaling, proliferation, and developmental functions, including Fc epsilon RI signaling, cholinergic synapse, rap1 signaling, axonogenesis, and hallmark G_2_-M checkpoint (Fig. 3B). Notably, cSCLC-LCNEC showed the highest enrichment of metabolic activities, including short-chain fatty acid metabolism, 2-oxobutyrate catabolic process, biotin metabolism, and hallmark oxidative phosphorylation (Fig. 3B).

Protein–protein interaction (PPI) network analyses were conducted to explore the molecular pathway relationships between subtype-specific proteins and key molecular players within each pathway (Fig. 3C). Further analysis focused on 2 key pathways: the LCNEC-specific antigen processing and presentation pathway (6 proteins) and the SCLC-specific rap1 signaling pathway (5 proteins), both of which are critical for the immune response and cancer development, proliferation, and invasion, respectively (Fig. 3D).

We used the enumerated combination method for histology-specific proteins and identified 2 proteins (NEU1 and SERPINB1) as potential diagnostic panels to discriminate between LCNEC and SCLC (Fig. S8). This 2-protein panel showed an area under the curve (AUC) of 0.938 [95% confidence interval (CI), 88.7% to 98.8%] in the CHCAMS cohort and an AUC of 0.814 (95% CI, 68.3% to 94.5%) in another independent immunohistochemistry (IHC) validation cohort (Fig. 3E). The representative cases of these 2 protein biomarkers observed in LCNEC and SCLC were shown in Fig. 3F.

### Immunological and metabolic characterization of Lu-NECs

**Fig. 4.**
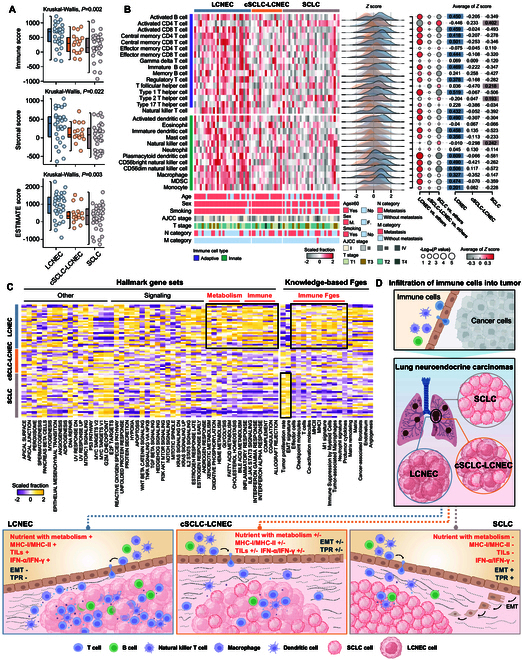
Different immune and metabolic characteristics among Lu-NECs. (A) Box plots showing the different distribution of immune score, stromal score, and ESTIMATE score among 3 Lu-NEC subtypes. *P* values were calculated with the Kruskal–Wallis test (*P* < 0.05). (B) Heatmaps showing the distribution of tumor-infiltrating immune cells among LCNEC, cSCLC-LCNEC, and SCLC. Dot plots showing the difference of immune cells in Lu-NECs and *P* values were calculated with the Kruskal–Wallis test (among 3 clusters) and the Wilcoxon test (between 2 clusters). (C) Heatmaps showing the distribution of enrichment scores of hallmark gene sets and knowledge-based Fges between LCNEC, cSCLC-LCNEC, and SCLC. (D) Schematic representation of the immune-desert and immune-inflamed tumors between LCNEC, cSCLC-LCNEC, and SCLC.

To decipher the immunological characteristics of Lu-NECs, we performed ssGSEA and ESTIMATE analysis of the proteomic data to infer the relative abundance of different immune and stromal cell types in the tumor microenvironment (TME) (Table S3). The LCNEC subtype showed a significant correlation with "immune hot" characteristics, as indicated by the highest immune score, adaptive immune fraction and innate immune fraction, and increased fractions of various immune cell types compared to cSCLC-LCNEC and SCLC (Kruskal–Wallis test *P* < 0.05; Fig. 4A and B and Fig. S9A to C). The immunological features of the transcriptomic datasets were also robustly reproduced in Lu-NECs (LCNEC, *n* = 66 and SCLC, *n* = 81) (Fig. S9D).

Based on the above results, we further investigated the metabolic and signaling alterations to explore the interplay between the immunologically hot LCNEC and other Lu-NEC phenotypes. Notably, LCNEC revealed a significant enrichment of immune-related and metabolism-related gene sets from both hallmark and knowledge-based functional gene expression signatures (Fges) (Fig. 4C). Conversely, SCLC was associated with increased tumor proliferation rate, epithelial–mesenchymal transition (EMT) signature, G_2_-M checkpoint, and E2F targets (Fig. 4C and Fig. S10A). We further investigated the relationship between immune cell infiltration and immune- and metabolism-related gene sets. Protumor cytokines, major histocompatibility complex class I (MHC-I), and MHC-II were positively correlated with immune cell infiltration in Lu-NECs, except for activated CD4^+^ T cells (Fig. S10B; *P* < 0.05 with Spearman's rank correlation). The proportion of immune infiltrated cells was predominantly negatively associated with increased tumor proliferation rate, EMT signature, G_2_-M checkpoint, and E2F targets.

Given the immunosuppressive effects of EMT signature, tumor proliferation rate, E2F targets, and G_2_-M checkpoint in response to interferon and inflammation, we further investigated the activation of interferon and inflammation in different Lu-NEC subtypes. As shown in Fig. S10C, the proportion of interferon-α (IFN-α) response, IFN-γ response, inflammatory response, and interleukin-6 (IL-6)–Janus kinase (JAK)–signal transducer and activator of transcription 3 (STAT3) signaling was highest in the immunologically hot LCNEC compared to the other immunologically cold subtypes (Kruskal–Wallis test, *P* < 0.05). These results suggest that LCNEC was characterized by an activated immune microenvironment and enhanced nutrient metabolism, whereas SCLC was predominantly associated with tumor proliferation (Fig. 4D).

### Disease progression-associated proteomic alterations for oncogenic pathways

**Fig. 5.**
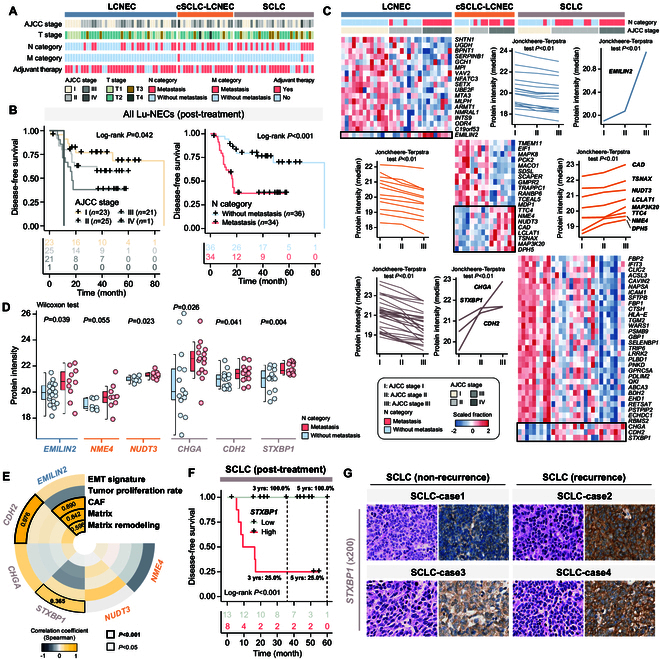
Proteogenomic alterations associated with disease progression for oncogenic pathways. (A) Distribution of clinicopathological characteristics in Lu-NECs. (B) Kaplan–Meier curves showing the different prognosis among the AJCC stage and N category. *P* values were calculated with the log-rank test. (C) Heatmaps showing the distribution of trending proteins from AJCC-I to AJCC-III in LCNEC, cSCLC-LCNEC, and SCLC, respectively. *P* values were calculated with the Jonckheere–Terpstra test. (D) Box plots showing the difference of the trending proteins in LCNEC (blue), cSCLC-LCNEC (yellow), and SCLC (brown), respectively. (E) Association between the intensity of trending proteins and the enrichment scores of tumor proliferation and invasion. *P* values were calculated with Spearman's rank correlation coefficient test. (F) Kaplan–Meier curves showing the difference in prognosis between patient groups with high and low *STXBP1* protein intensity. The *P* value was calculated with the log-rank test. (G) Representative H&E and *STXBP1* IHC staining images (200× magnification) in SCLC.

Our analysis of clinicopathological features, such as American Joint Committee on Cancer (AJCC) and tumor–node–metastasis (TNM) stages, revealed an unbiased distribution across histological types of Lu-NECs (Fisher's exact tests, *P* > 0.05; Fig. 5A). Compared to the early AJCC stage and non-lymph node metastatic group, the advanced and metastatic group showed a significantly poor prognosis (Fig. 5B and Fig. S11A). Meanwhile, a spectrum of protein intensity changes was observed in Lu-NECs across different AJCC stages and metastatic groups (Fig. S11B). Monotonic trend analyses from AJCC stages I to III identified 18, 20, and 33 differentially expressed proteins in LCNEC, cSCLC-LCNEC, and SCLC, respectively (Jonckheere–Terpstra test, *P* < 0.01). Among them, 1 protein in LCNEC, 8 in cSCLC-LCNEC, and 3 in SCLC showed a monotonic increase in intensity, while the remaining 17 in LCNEC, 12 in cSCLC-LCNEC, and 30 in SCLC showed a monotonic decrease (Fig. 5C). Functional enrichment analyses of differentially expressed proteins with monotonic trends revealed significant enrichment in immune- and metabolism-related functions in LCNEC, including B cell/T cell receptor signaling and nucleotide-sugar metabolism processes (Fig. S12A). Conversely, SCLC was characterized by dysregulation in pathways related to myeloid leukocyte-mediated immunity, the PPP, and neurotransmitter signaling (Fig. S12A), with cSCLC-LCNEC showing complex enrichments of differentially expressed proteins with monotonic trends (Fig. S12A).

To further explore the differential protein intensity in cancer progression and invasion, we analyzed the metastatic and nonmetastatic groups, focusing on the above trend proteins, and found that EMILIN2 is a critical protein in LCNEC, while NUDT3 and NME4 are key players in cSCLC-LCNEC. In SCLC, STXBP1, CDH2, and CHGA were identified as critical proteins (Fig. 5D). Correlation analyses between protein intensities and cancer-related pathways further elucidated the roles of these key proteins in Lu-NEC growth and metastasis (Fig. 5E). In particular, EMILIN2 in LCNEC showed significant positive correlations with cancer-associated fibroblasts (CAFs), matrix, and matrix remodeling (Spearman's *r* values of 0.890, 0.842, and 0.596, respectively; *P* < 0.001). Similarly, significant associations were found between the EMT signature and CDH2 (Spearman's *r* = 0.976, *P* < 0.001) and STXBP1 (Spearman's *r* = 0.365, *P* = 0.047) in SCLC (Fig. S12B). Furthermore, our analyses revealed that increased intensity of STXBP1, EMILIN2, and CDH2 was associated with poor prognosis in LCNEC and SCLC (Fig. 5F and Fig. S12C and D). Specifically, higher STXBP1 expression correlated with significantly worse prognosis (log-rank *P* < 0.001), and no recurrence events were observed in the lower STXBP1 expression group in SCLC (Fig. 5F). The STXBP1 expression was also able to predict the recurrence of SCLC patients [AUC–receiver operating characteristic (ROC) = 0.922] compared to CDH2 (AUC–ROC = 0.633; Fig. S12D). IHC analysis of pathological sections also confirmed that STXBP1 expression was 3.5-fold higher in SCLC recurrence samples (median H-score: 143.585 versus 41.24) than in nonrecurrence samples (Fig. 5G and Fig. S12E).

## Discussion

Recent advances in genomic and transcriptomic analyses of LCNEC and SCLC have improved our understanding of the molecular features of Lu-NECs [16,19–21]. Despite these advances, there remains a critical need to delve deeper into the complex tumor biology of Lu-NECs from a proteogenomic perspective. Here, we performed a comprehensive proteogenomic profiling of Lu-NECs in the Chinese population, contributing a valuable resource and complementary insights into the complex biology of Lu-NECs. To the best of our knowledge, this is the initial investigation to systematically unravel the proteomic characteristics of different histological subtypes in Lu-NECs, providing scientific evidence and clinical translational potential for further understanding and seeking potential diagnostic and therapeutic targets in Lu-NECs.

The comparative analysis of genomics and proteomics distinguishing between LCNECs, SCLCs, and cSCLC-LCNECs delineates a complex spectrum of shared and disparate molecular alterations and aberrant signaling pathways that contribute to the unique biological and clinical behaviors of the different histological subtypes of Lu-NECs. Consistent with previous genomic studies [9,16,22–24], we observed high mutation loads and transversions in Lu-NECs, indicative of tobacco exposure [23,25]. Interestingly, TMB and *LRP1B* mutation frequencies showed similar trend fluctuations in Lu-NECs, suggesting that *LPR1B* mutations correlate with high TMB status and may serve as potential predictive indicators for favorable outcomes of immunotherapies in lung cancer [26–28]. Besides, genomic stability measures, including wGII and MSI scores, did not show significant disparities between subtypes, suggesting that these factors alone may not fully account for the observed clinical heterogeneity. However, the differential CNV burden, particularly between SCLC and the other subtypes, highlights potential differences in genomic regulation and tumor evolution. The integrated analysis of genomics and proteomics enabled us to link genotype to phenotype and investigate the consequences of genomic variation on cellular physiology. Although previous studies have consistently reported pervasive *RB1* mutations in SCLCs and LCNECs, our proteogenomic analysis revealed different *RB1* mutation rates and their consequential effects in different Lu-NECs subtypes, implying distinct impacts on protein expression and clinical prognosis. Specifically, we demonstrated a correlation between *RB1* mutations and reduced *RB1* protein intensity, leading to unfavorable outcomes in cSCLC-LCNEC.

The TME plays a critical role in cancer progression and is composed of tumor cells and various nonmalignant cell types, predominantly immune cells [29–31]. The complex immunoregulatory processes and metabolic crosstalk within the TME significantly influence cancer progression and metastasis [32–36]. Previous studies have delineated an immunologically cold TME in SCLC, characterized by low T cell infiltration and low MHC class I expression, with only a few tumors exhibiting an inflamed phenotype [37–40]. However, little is known about the TME in LCNECs, particularly from a proteomic perspective. Functional enrichment analyses highlighted subtype-specific pathways, with LCNEC associated with immune and metabolic functions, and SCLC associated with signaling and proliferation, consistent with findings from other proteomic studies [41,42]. We hypothesized that LCNEC would be more sensitive to immunotherapy, consistent with the robust responses to checkpoint inhibitors observed in some stage IV LCNEC cases [43,44]. Moreover, the limited survival benefit (less than 20%) seen in extensive-stage SCLC patients treated with chemotherapy combined with immunotherapy could be attributed to their relatively cold immune microenvironment. Notably, cSCLC-LCNEC has the highest TMB, making it more eligible for immunotherapies such as immune checkpoint inhibitors (ICIs) [45,46].

Furthermore, our analysis highlighted an overrepresentation of metabolic pathways in LCNEC and cSCLC-LCNEC, contrasting with the limited metabolic activity in SCLC. LCNEC showed an up-regulated PPP, a major metabolic branch of glycolysis responsible for generating nicotinamide adenine dinucleotide phosphate (NADPH) and ribose sugars, essential for biosynthesis and mitigating oxidative stress in cancer cells [47,48]. Besides, as reported in previous studies [49,50], activation of T lymphocytes increases glucose consumption by the PPP, which correlates with our observations of increased immune cell infiltration in LCNEC. Our proteomic analyses shed light on the correlation between metabolism reprogramming and immunity within Lu-NECs. Specifically, we observed a pronounced enrichment of metabolic activities in cSCLC-LCNEC, especially in enhanced fatty acid metabolism. As a vital metabolic hallmark of cancer cells, deregulated fatty acid metabolism plays a pro-oncogenic role via membrane biogenesis, energy supply, and protein modification [51–55]. Additionally, fatty acid metabolism displayed a crucial role in the beginning of the tricarboxylic acid cycle, which was essential for tumor cell growth [55]. Several studies have demonstrated the vulnerability of SCLC to disruption of lipid metabolism [56–58], suggesting that targeting fatty acid metabolism could be a viable therapeutic strategy for cSCLC-LCNEC. More importantly, we found that cSCLC-LCNEC exhibited unique characteristics beyond the “mixed phenotypes” of LCNEC and SCLC, suggesting the occurrence of exclusive biological processes in the tumorigenesis and progression of cSCLC-LCNEC.

Accurate differentiation between SCLC and LCNEC is critical due to different treatment strategies required in clinical practice, despite their similar histological and immunohistochemical features [10–12]. Previous studies have identified heterogeneous genetic biomarkers with limited utility for diagnostic purposes [6,9,16]. Here, unsupervised clustering and differential intensity analyses delineated distinct proteomic profiles among Lu-NEC subtypes. This segregation corroborated our genomic findings and provided an additional layer of molecular differentiation that could be leveraged for diagnostic and therapeutic purposes. Specifically, we identified and validated a panel of 2 proteins that provide ancillary diagnostic tests. In addition, we discovered critical proteins involved in cancer progression and invasion within different Lu-NECs, which held significant potential for developing protein biomarkers for disease progression and prognosis prediction.

Several limitations need to be addressed. First, this study is a retrospective resection cohort study, and further validation is needed in advanced cases. Second, this study focuses on the comparison between tumors in Lu-NECs and does not consider nontumor tissues. Third, protein cross-linking in FFPE samples may lead to the masking of certain epitopes, resulting in the loss of expression of some proteins. Last, the identified potential biomarkers require further investigation in vivo or in vitro, although coculture of the cSCLC-LCNEC cell lines remains challenging.

## Conclusion

Our study provides a comprehensive genomic and proteomic landscape of Chinese Lu-NECs and deciphers the similarities and differences of molecular and functional features among different histological subtypes. Furthermore, our results highlight the importance of subtype-specific proteins in refining diagnostic accuracy, prognostic assessment, and developing personalized therapeutic approaches. This study significantly advances our understanding of Lu-NEC biology and provides an important resource for advancing both basic and clinical research in this field.

## Methods

### Study design and patient samples

This study included FFPE tumor samples from patients with operable Lu-NECs who underwent surgical resection and were recruited between 2011 and 2021 from the Cancer Hospital of the Chinese Academy of Medical Sciences, National Cancer Center of China. Patients were randomly divided into 2 cohorts: a discovery cohort (proteogenomic cohort, *n* = 93) and a validation cohort (IHC cohort, *n* = 59). Each FFPE sample was reviewed by 2 experienced lung cancer pathologists (X.S. and L.Y.), who examined the pathology slides, verified the diagnoses, and selected the most representative blocks. The serial sections were then cut from the whole block and sent for analysis. During the analysis, experimental quality control was strictly followed. Tumor areas were outlined, and nontumor tissues, such as lung parenchyma and bronchial cartilage, were excluded to eliminate the influence of normal components.

After quality control, 93 cases (42 LCNEC, 21 cSCLC-LCNEC, and 30 SCLC) underwent WES and 76 cases (including 30 LCNEC, 16 cSCLC-LCNEC, and 30 SCLC) underwent proteomic sequencing in the discovery cohort. All 59 cases (including 29 LCNEC and 30 SCLC) in the validation cohort were subject to IHC staining verification for protein biomarkers. Survival was followed until October 2022. Tissues were all postoperative FFPE samples, each with sufficient material, complete clinical and follow-up data, primarily including disease-free survival (DFS) and overall survival (OS), with diagnoses confirmed by 2 experienced pathology professors. The clinical information of these enrolled patients is shown in Table S1.

### WES data processing

Methods are detailed in Supplementary Methods.

### Proteomic data processing

Methods are detailed in Supplementary Methods.

### Public Lu-NEC patient cohorts

The independent cohorts of Lu-NEC patients with accessible RNA-sequencing (RNA-seq) data and clinical information were obtained from the supplementary profiles of relevant publications and the Gene Expression Omnibus database (GEO), including 66 LCNEC patients from the George (2018) cohort [9], 81 SCLC patients from the George (2015) cohort [22] and 78 SCLC patients from the Jiang cohort [59]. The read count of LCNEC RNA-seq data (George, 2018) was quantified using RNA-seq by expectation-maximization (RSEM), while the expression of SCLC RNA-seq data (George, 2015) was measured using fragments per kilobase of transcript per million mapped reads (FPKM) values. The read count of the Jiang cohort was transformed using log_2_(*x*).

### Immunohistochemistry

Tissue microarray (TMA) blocks were prepared from representative paraffin-embedded tissues selected by the consultant pathologist, 1.5 mm in diameter (2 cores/paraffin tissue). IHC was performed on 4-μm-thick FFPE TMA sections using fully automated Roche immunohistochemistry instruments [Bench-Mark ULTRA IHC/ISH System, Roche Diagnostics, Bench-Mark ULTRA System (roche.com)] according to standard protocols. The following primary antibodies were used: *STXBP1* (11459-1-AP, Proteintech, 1:100 dilution), *NEU1* (67032-1-Ig, Proteintech, 1:200 dilution), and *SERPINB1* (ab47731, Abcam, 1:100). After antigen retrieval and peroxidase blocking, sections were incubated with primary antibodies, followed by horseradish peroxidase (HRP)-conjugated secondary antibodies and 3,3'-diaminobenzidine (DAB) chromogen for visualization. Counterstaining was performed with hematoxylin. *STXBP1*, *NEU1*, and *SERPINB1* stainings were all located in the cytoplasm. Digital scans of the stained sections were analyzed using QuPath software v0.3.2 (https://qupath.github.io), an open-source digital pathology image analysis software, integrating staining intensity and cell positivity percentages to calculate the H-scores under 200× field microscopy. Controls were included to ensure validity, and a dual pathologist review was conducted to ensure analytical accuracy.

### Differential analysis of protein intensities

Differential analysis of protein intensities was performed using the Wilcoxon rank-sum test and adjusted for multiple comparisons using the false discovery rate (FDR) method. Proteins were considered significantly differentially expressed with an adjusted *P* value of <0.1 and a fold change (FC) of >1.5 (or FC < 2/3).

### Gene set and pathway enrichment analysis

The knowledge-based Fges and hallmark gene sets were obtained from a previous study [60] and the MSigDB database (https://www.gsea-msigdb.org/gsea/msigdb). The histological subtype-specific up-regulated proteins were used to perform the enrichment analysis on the Gene Ontology (GO) biological process (BP), Kyoto Encyclopedia of Genes and Genomes (KEGG) pathway, and hallmark gene sets using the hypergeometric test based on the “clusterProfiler” (v.4.8.3) R package. *P* values were adjusted with the FDR method. Single-sample GSEA (ssGSEA) was performed to calculate the score of Fges and hallmark gene sets based on global proteomic data using the “GSVA” (v.1.48.3) R package.

### PPI network

The PPI networks were assessed and visualized from the STRING database (v.12.0) [61]. For each Lu-NEC, the proteins acquired in the same gene set enrichment were mapped to PPI networks, with the removal of the singular protein node.

### Estimation of stromal and immune scores and proportion of tumor-infiltrating immune cells

Stromal and immune scores were estimated using the ESTIMATE (v.1.0.13) R package based on proteomic data [62]. The proportions of 28 immune cell signatures [63], immune-related Fges [60], and immune-related hallmark gene sets were calculated using the ssGSEA method with “GSVA” (v.1.48.3) R package based on proteomic data, as described in previous studies [64,65].

### Statistical analysis

All statistical tests were performed using R software (v.4.1.3), and *P* values were adjusted for multiple comparisons using the FDR method. Unless otherwise stated, comparisons of continuous variables were performed using the unpaired Wilcoxon rank sum test or the Kruskal–Wallis test on 2 or 3 groups, separately. Fisher’s exact tests were used to compare categorical distributions. The correlation coefficient between continuous variables was used in Spearman’s correlation test. The Jonckheere–Terpstra test was used to quantify trend variation in continuous variables between groups in the “clinfun” (v.1.1.5) R package. Univariate and multivariate Cox proportional hazards regression analyses were performed using the “survival” (v.3.4-0) R package, and hazard ratio (HR) and 95% CI were calculated. Differences in survival were compared between 2 or more groups using Kaplan–Meier survival curves and log-rank tests with the “survminer” (v.0.4.9) R package. The diagnostic performance of the proteomics-based model was evaluated using the ROC curve and the AUC. Delong’s tests were performed to calculate the significance between different AUCs using the “pROC” (v.1.18.5) R package.

## Ethical Approval

This retrospective study was approved by the Ethics Committee and Institutional Review Boards of the National GCP Center (approval no. 22/250-3452), and all patients were exempted from informed consent due to the retrospective nature of this study.

## Data Availability

The WES data generated in this study have also been deposited in GSA-human (https://ngdc.cncb.ac.cn/gsa-human/) under accession code HRA007102, and the mass spectrometry proteomic data generated in this study have also been deposited in OMIX (https://ngdc.cncb.ac.cn/omix/) under accession code OMIX006183, in accordance with the necessary approval from the Chinese Ministry of Science and Technology related to export the genetic information and materials associated with this study. Other public Lu-NEC cohorts with transcriptomic data and clinical information are available from the publications and supplementary information from George’s study (2015 and 2018) and Jiang’s study. The source code of this work can be downloaded from https://github.com/ZhoulabCPH/Proteogenomic_Lung_NECs.git.

## Supplementary Materials

Supplementary 1 Supplementary Methods Figs. S1 to S12 Tables S1 to S3 References
