# Supplementary material for: Proteogenomic Characterization of High-Grade Lung Neuroendocrine Carcinoma Deciphers Molecular Diversity and Potential Biomarkers of Different Histological Subtypes in Chinese Population
Source: Research (Wash D C). 2025 Apr 14;8:0671. doi: 10.34133/research.0671 (PMC11994885; doi:10.34133/research.0671)
Supplement: Supplementary 1 — Supplementary Methods Figs. S1 to S12 Tables S1 to S3 References [file research.0671.f1.zip › Supplementary information.docx]

**Supplementary Methods**

**Whole-exome sequencing data processing**

***DNA extraction.*** DNA was extracted from FFPE slides following the protocols outlined in the manufacturer's instructions for the Maxwell 16 FFPE Plus LEV DNA Purification Kit (Promega, Madison, WI, USA, #AS1135). The integrity and purity of both isolated and potentially contaminated samples were rigorously assessed using the following methods: (i) DNA degradation and contamination were examined by electrophoresis on 1% agarose gels; (ii) DNA concentration was quantified using the Qubit® DNA Assay Kit on a Qubit® 3.0 Fluorimeter (Invitrogen, CA, USA).

***DNA library construction and sequencing.*** 0.2 μg of genomic DNA per sample was used as the starting material for DNA preparation. Sequencing libraries were carefully constructed using the Agilent SureSelect Human All Exon V8 Kit (Agilent Technologies, CA, USA) per the manufacturer's guidelines. Unique index codes were then assigned to each sample. Briefly, DNA fragmentation was performed using a hydrodynamic shearing system (Covaris, Massachusetts, USA) to generate fragments of 180-280 base pairs. The remaining overhangs were effectively converted to blunt ends by exonuclease and polymerase activities. After adenylating the 3' ends of the DNA fragments, adapter oligonucleotides were ligated. DNA fragments with adapter molecules ligated at both ends were selectively enriched by polymerase chain reaction (PCR). Following the PCR reaction, the libraries were hybridized to a liquid phase using a biotin-labeled probe. Streptomycin-coated magnetic beads were then used to capture the exonic regions of the genes. The captured libraries were enriched by a further PCR reaction to incorporate index tags in preparation for sequencing. The resulting products were purified using the CleanNGS system (CleanNA, Netherlands) and quantified using the Agilent high-sensitivity DNA assay on the Agilent Bioanalyzer 4200 system. The DNA libraries were sequenced on the Illumina Novaseq 6000 platform, resulting in 150 base pair paired-end reads.

***RAW data quality control.*** PE150 sequencing was used for each sample. However, the length of DNA segments may be less than 150 base pairs due to the presence of adapter sequences in the raw reads. Fastp (v. 0.22.0) was used to remove adapter sequences from the raw Fastq data. Reads with lengths greater than 75 base pairs were selected for further analysis.

***Reads mapping to the reference sequence.*** High-quality reads were aligned to the UCSC human reference genome (hg19) using the Burrows-Wheeler Aligner (BWA, v. 0.7.12) software MEM (maximal exact matched) algorithm with default parameters. We then obtained the original mapping results, and sorted and formatted them into a BAM file. PCR duplicates in the BAM files were flagged, regions potentially containing indel mutations were realigned, and the quality of each base pair was recalibrated. The analysis primarily involved the assessment of several key metrics, including the ratio of bases at different depths (1x, 10x, 20x) compared to the reference genome, PCR redundancy and sequencing depth. These metrics played a central role in assessing the quality and depth of the sequencing data.

***Somatic mutation detection.*** SNV and InDel detection was performed in each tumor sample using Sentieon's TNseq (v. 2020.10.01) in tumor-only mode. Mutations in low complexity regions, including tandem repeats and highly homologous regions, were systematically excluded. Low-confidence variants were excluded if any of the following conditions were not met: (i) total depth greater than 10; (ii) alternative allele depth greater than 3; (iii) splice sites within 5 bases; (iv) and variant allele fraction (VAF) greater than 0.05 (1). In addition, discrimination of germline variation was improved by reference to population allele frequencies. These references were derived from gnomAD and a panel of normal (PON) derived from healthy blood samples. As a final step, all mutations meeting high confidence criteria were annotated using ANNOVA (v.2018.04.16). To reduce the influence of DNA oxidative damage in FFPE samples (2), we systematically filtered out C>A (G>T) and C>T (G>A) mutations with a VAF less than 0.25, resulting in the number of C>A and C>T mutations approximately equal to that of G>T and G>A mutations for further analysis (1).

***Copy number variant detection.*** CNV detection within the genome was performed using the CNVkit software(3). A bin size of 5 kb was used to analyze the whole gene copy number variation comprehensively. Initially, copy number coverage (CNC) data were calculated for each bin size of the sample. The CNC file was then compared with the reference sequence. In parallel, corrections for bias and GC content were applied to derive a copy number ratio file for a sample. Further refinement was achieved using the Circular Binary Segmentation (CBS) algorithm(4). This method linked the copy number ratio file segments with similar log2 values to bin size, providing insight into copy number changes within different segment regions. Regions with absolute log2 copy number ratios greater than 0 indicated gains (amplifications), while those with ratios less than 0 indicated losses (deletions).

**Proteomic data processing**

***Protein extraction and trypsin digestion.*** Samples were deparaffinized with xylene. Four volumes of lysis buffer (containing 1% SDS and 1% protease inhibitor cocktail) were then added to the tissue. This was followed by three minutes of sonication on ice using a high-intensity ultrasonic processor, specifically the one manufactured by Scientz. Residual debris was removed by centrifugation at 12,000g for 10 minutes at 4°C. Finally, the resulting supernatant was carefully collected, and the protein concentration was quantified using the BCA kit according to the manufacturer's instructions. The protein sample was combined with 1 volume of pre-cooled acetone, followed by thorough vortexing to ensure proper mixing. Four volumes of pre-cooled acetone were then added, resulting in precipitation at a temperature of -20°C for 2 hours. The resulting precipitate was washed 2-3 times with pre-cooled acetone. The protein sample was then reconstituted in a solution of 200 mM TEAB (triethylammonium bicarbonate) and dispersed by ultrasound. For initial digestion, trypsin was added at a trypsin-to-protein mass ratio of 1:50 and digestion was allowed overnight. The sample was first reduced with 5 mM dithiothreitol at 56°C for 30 minutes and then alkylated with 11 mM iodoacetamide for 15 minutes at room temperature without light. The resulting peptides were then purified and desalted on a Strata X Solid Phase Extraction (SPE) column.

***LC-MS/MS analysis.*** The tryptic peptides were reconstituted in solvent A and then loaded directly onto a custom-made reversed-phase analytical column (25 cm length, 100 μm i.d.). The mobile phase was a mixture of solvent A (containing 0.1% formic acid and 2% acetonitrile in water) and solvent B (containing 0.1% formic acid and 90% acetonitrile in water). The peptides were carefully separated using the following gradient elution profile: 0-22.5 min, 6%-22%B; 22.5-26.5 min, 22%-34%B; 26.5-28.5 min, 34%-80%B; 28.5-30 min, 80%B. Chromatography was performed at a constant flow rate of 700 nanoliters per minute on a ThermoFisher Scientific EASY-nLC 1200 UPLC system. The isolated peptides were analyzed on an Orbitrap Exploris 480 mass spectrometer with a nano-electrospray ion source. An electrospray voltage of 2300 volts was used for ionization. The high-field asymmetric waveform ion mobility spectrometry (FAIMS) compensation voltage (CV) was set to '-'. Both precursor and fragment ions were analyzed using the Orbitrap detector. For the full MS scan, a resolution of 60,000 was selected, covering a mass-to-charge (m/z) range of 350-1400. For the MS/MS scan, the first mass was fixed at 120.0 m/z and analyzed at a resolution of 15,000. High Energy Collision Dissociation (HCD) fragmentation was performed with a normalized collision energy (NCE) of 27%. The automatic gain control (AGC) target was set to 1^6 and the maximum injection time was limited to 22 milliseconds.

***MS database search.*** DIA data were processed using the DIA-NN search engine (v.1.8). Tandem mass spectra were searched against a FASTA data set (consisting of 20,389 entries) concatenated with an inverted decoy database. Trypsin/P was the cleavage enzyme, allowing for up to 1 missing cleavage. Fixed modifications were defined as excision at the N-terminal Met and carbamidomethyl at Cys. The false discovery rate (FDR) was carefully controlled and adjusted to less than 1% to ensure data quality.

***Proteomic data normalization.*** A total of 9,707 proteins (containing at least one unique peptide segment) were identified after searching the MS database. Proteins identified in less than 75% of the samples were filtered out and transformed using log2(x) for subsequent analyses. Missing values in the filtered protein profile were imputed using DreamAI (https://github.com/WangLab-MSSM/DreamAI). These approaches have been used consistently in other published proteomic studies(1, 5-9).

***Sample batch effect and quality control.*** The protein profiles of all Lu-NEC samples were from the same sequencing batch. Spearman's correlation coefficients were calculated for the association between each two samples after normalization. The density for the normalized protein intensities of each sample showed the same unimodal distribution across Lu-NEC subtypes.

**Estimation of weighted genome instability index, microsatellite instability score and copy number variation burden**

***Weighted genome instability index (wGII).*** The weighted Genomic Instability Index (wGII) was calculated by determining the average proportion of the genome displaying aberrant copy number, with each of the 22 autosomal chromosomes (excluding the X and Y chromosomes) being assigned the weight (10).

***Microsatellite instability score (MSI) score.*** The MSI score (%) was calculated using the published method ‘MSIsensor2’ (https://github.com/niu-lab/msisensor2.git). The MSIsensor2, a machine learning-based tool, was implemented to analyze sequencing data obtained exclusively from FFPE tumor samples.

***Copy number variation burden (CNV burden).*** The CNV burden for each sample was determined by the following calculation: the number of bases within genomic segments with a log2 CN ratio greater than 0.1 or less than -0.1 was divided by the total number of bases across all segments in the genome.

***Cis-* and *trans-*effects of genetic driver alterations on the proteome**

The knowledgeable list of 568 cancer driver genes was obtained from Francisco et al. (11). The *cis-*effects were defined as the effect of a specific mutation in a cancer driver gene on the expression or abundance of the protein produced by that gene. Conversely, *trans-*effects indicated the effect of driver mutations on protein levels that were not associated with the specific driver gene that was mutated. In essence, the *cis-* and *trans-*effects represented the direct influence of a mutation in a particular driver gene on the protein levels encoded by that gene. The Wilcoxon rank sum test was used to calculate the differential intensity of proteins between the mutant and wild-type groups.

**Supplementary References**

1. Geffen Y, Anand S, Akiyama Y, Yaron TM, Song Y, Johnson JL, et al. Pan-cancer analysis of post-translational modifications reveals shared patterns of protein regulation. Cell. 2023;186(18):3945-67.e26.

2. Chen L, Liu P, Evans TC, Jr., Ettwiller LM. DNA damage is a pervasive cause of sequencing errors, directly confounding variant identification. Science (New York, NY). 2017;355(6326):752-6.

3. Talevich E, Shain AH, Botton T, Bastian BC. CNVkit: Genome-Wide Copy Number Detection and Visualization from Targeted DNA Sequencing. PLoS computational biology. 2016;12(4):e1004873.

4. Hsu FH, Chen HI, Tsai MH, Lai LC, Huang CC, Tu SH, et al. A model-based circular binary segmentation algorithm for the analysis of array CGH data. BMC research notes. 2011;4:394.

5. Burns J, Wilding CP, Krasny L, Zhu X, Chadha M, Tam YB, et al. The proteomic landscape of soft tissue sarcomas. Nature communications. 2023;14(1):3834.

6. Li L, Jiang D, Zhang Q, Liu H, Xu F, Guo C, et al. Integrative proteogenomic characterization of early esophageal cancer. Nature communications. 2023;14(1):1666.

7. Niu L, Thiele M, Geyer PE, Rasmussen DN, Webel HE, Santos A, et al. Noninvasive proteomic biomarkers for alcohol-related liver disease. Nature medicine. 2022;28(6):1277-87.

8. Dong L, Lu D, Chen R, Lin Y, Zhu H, Zhang Z, et al. Proteogenomic characterization identifies clinically relevant subgroups of intrahepatic cholangiocarcinoma. Cancer Cell. 2022;40(1):70-87 e15.

9. Gillette MA, Satpathy S, Cao S, Dhanasekaran SM, Vasaikar SV, Krug K, et al. Proteogenomic Characterization Reveals Therapeutic Vulnerabilities in Lung Adenocarcinoma. Cell. 2020;182(1):200-25 e35.

10. Liang J, Sun G, Pan X, Zhang M, Shen P, Zhu S, et al. Genomic and transcriptomic features between primary and paired metastatic fumarate hydratase-deficient renal cell carcinoma. Genome medicine. 2023;15(1):31.

11. Martínez-Jiménez F, Muiños F, Sentís I, Deu-Pons J, Reyes-Salazar I, Arnedo-Pac C, et al. A compendium of mutational cancer driver genes. Nature reviews Cancer. 2020;20(10):555-72.

**Supplementary Tables**

**Supplementary Table 1. Baseline participant characteristics in this study**

| **Characteristics** | **LCNEC**  **N=42** | **cSCLC-LCNEC**  **N=21** | **SCLC**  **N=30** |
| --- | --- | --- | --- |
| **Age (years)** |  |  |  |
|  | 62 (60~65) | 62 (61~65) | 62 (54~67) |
| **Age [n (%)]** |  |  |  |
| >60 | 27 (64.3%) | 17 (81.0%) | 17 (56.7%) |
| ≤60 | 15 (35.7%) | 4 (19.0%) | 13 (43.3%) |
| **Tumor location [n (%)]** |  |  |  |
| Left | 17 (40.5%) | 7 (33.3%) | 13 (43.3%) |
| Right | 25 (59.5%) | 14 (66.7%) | 17 (56.7%) |
| **Tumor size (cm)** |  |  |  |
|  | 3.9 (2.0~5.0) | 3 (2.5~4.5) | 3.5 (2.7~4.5) |
| **Sex [n (%)]** |  |  |  |
| Male | 41 (97.6%) | 21 (100.0%) | 23 (76.7%) |
| Female | 1 (2.4%) | 0 (0.0%) | 7 (23.3%) |
| **Smoking [n (%)]** |  |  |  |
| Yes | 40 (95.2%) | 21 (100.0%) | 19 (63.3%) |
| No | 2 (4.8%) | 0 (0.0%) | 11 (36.7%) |
| **AJCC stage [n (%)]** |  |  |  |
| I | 18 (42.9%) | 7 (35.0%) | 9 (30.0%) |
| II | 13 (30.9%) | 6 (30.0%) | 13 (43.3%) |
| III | 11 (26.2%) | 7 (35.0%) | 7 (23.3%) |
| IV | 0 (0.0%) | 1 (5.0%) | 1 (3.3%) |
| **T stage [n (%)]** |  |  |  |
| T1 | 12 (28.6%) | 6 (28.6%) | 7 (23.3%) |
| T2 | 24 (57.1%) | 11 (52.4%) | 14 (46.7%) |
| T3 | 6 (14.3%) | 2 (9.5%) | 9 (30.0%) |
| T4 | 0 (0.0%) | 2 (9.5%) | 0 (0.0%) |
| **N category [n (%)]** |  |  |  |
| Metastasis | 14 (33.3%) | 11 (52.4%) | 16 (53.3%) |
| Without metastasis | 28 (66.7%) | 10 (47.6%) | 14 (46.7%) |
| **M category [n (%)]** |  |  |  |
| Metastasis | 0 (0.0%) | 1 (4.8%) | 1 (3.3%) |
| Without metastasis | 42 (100.0%) | 20 (95.2%) | 29 (96.7%) |
| **Adjuvant therapy [n (%)]** |  |  |  |
| Yes | 32 (76.2%) | 17 (81.0%) | 21 (70.0%) |
| No | 10 (23.8%) | 4 (19.0%) | 9 (30.0%) |
| **OS status [n (%)]** |  |  |  |
| Dead | 14 (33.3%) | 10 (47.6%) | 4 (13.3%) |
| Alive | 28 (66.7%) | 11 (52.4%) | 26 (86.7%) |
| **DFS status [n (%)]** |  |  |  |
| Recurrence | 21 (50.0%) | 13 (61.9%) | 10 (33.3%) |
| Non-recurrence | 21 (50.0%) | 8 (38.1%) | 20 (66.7%) |

**LCNEC**: Large cell neuroendocrine carcinoma; **cSCLC-LCNEC**: Combined small cell lung cancer and large cell neuroendocrine carcinoma; **SCLC**: Small cell lung carcinoma. **OS**: Overall survival; **DFS**: Disease-free survival.

**Data is displayed as median (interquartile range).**

**Values presented as n (%).**

**Supplementary Table 2. List of wGII, MSI score (%) and CNV burden in Lu-NECs.**

**Supplementary Table 3. The fraction of ESTIMATE and 28 immune cells in Lu-NECs.**

**Supplementary Figures:**

**
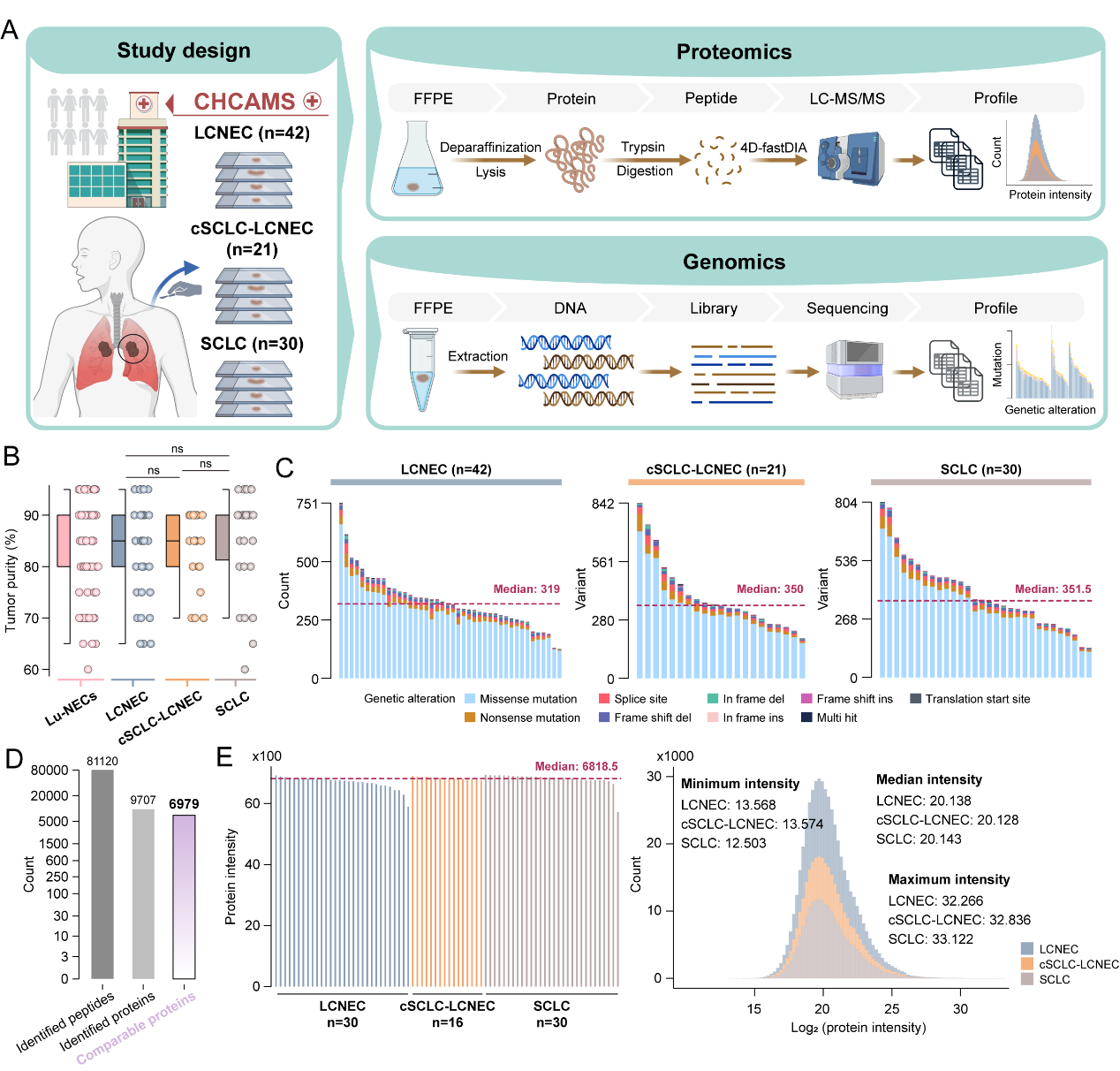
**

**Supplementary Figure 1. Proteogenomic landscape of high-grade lung neuroendocrine carcinoma.** (A) Schematic overview of the experimental design and proteogenomic atlas. (B) Box plots showing the percentage of tumor cells in H&E specimens based on the whole exome sequencing data (n=93). (C) Bar plots showing the number of genomic alterations between LCNEC, cSCLC-LCNEC and SCLC. The median number of genomic alterations is indicated by the red line. (D) Bar plot showing peptide yields, identified proteins and comparable proteins based on LC-MS analysis (n=76). (E) Bar plot (left panel) and stacked plot (right panel) showing the distribution of protein intensity in each sample among Lu-NECs. The median protein intensity is indicated by the red line.


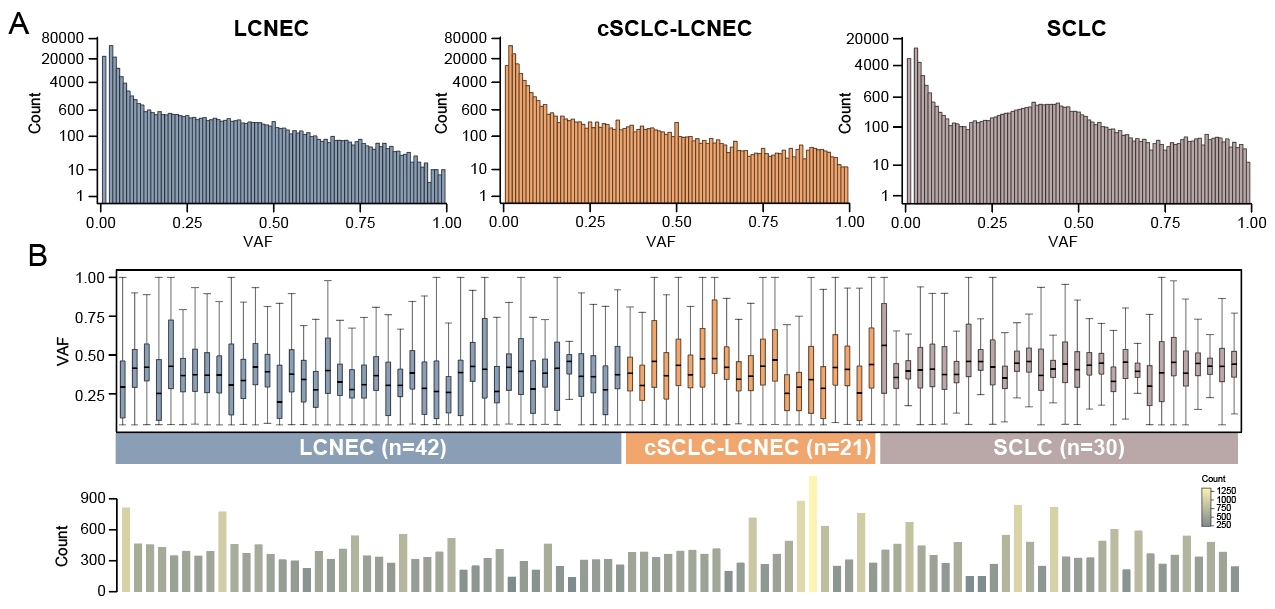


**Supplementary Figure 2. Overview of the distribution of somatic alterations in the CHCAMS cohort.** (A) Bar plots showing the distribution of variant allele frequencies (VAF) in LCNEC, cSCLC-LCNEC and SCLC. (B) Box plots and bar plots showing the distribution and number of high-quality somatic variants.


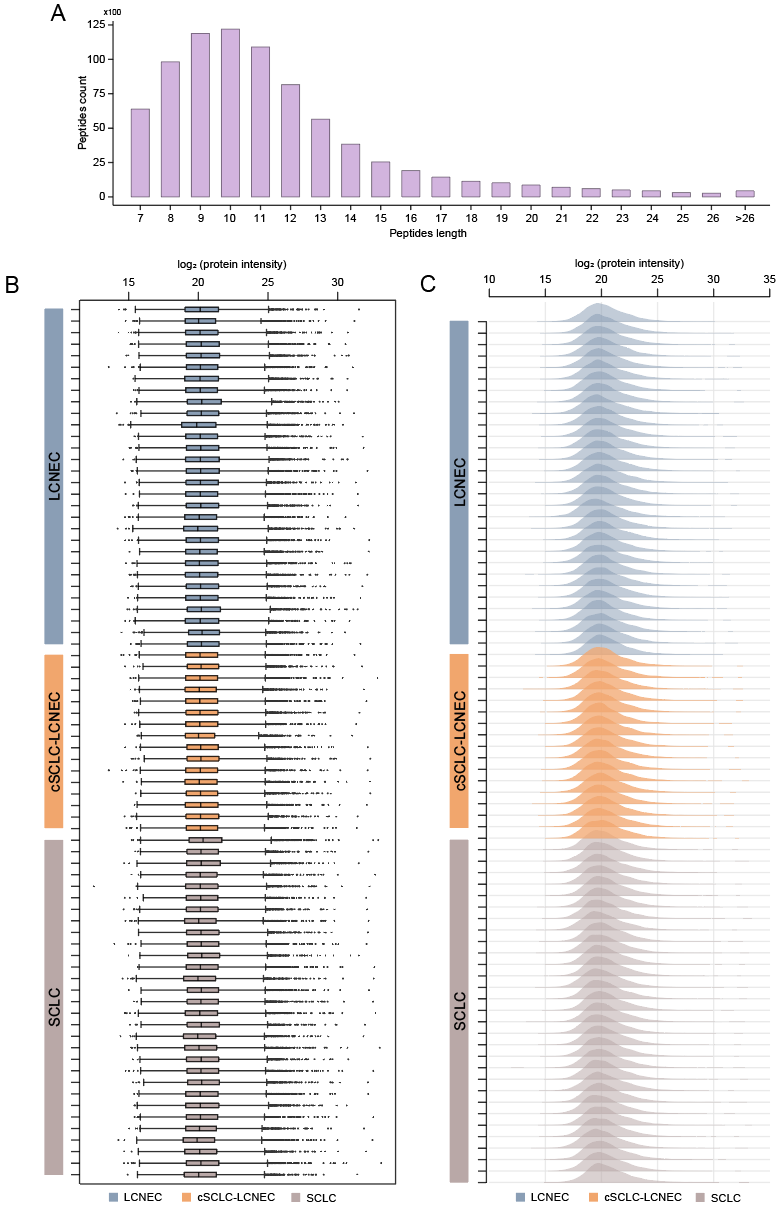


**Supplementary Figure 3. Overview of the protein intensity distribution in the CHCAMS cohort.** (A) Bar plots showing the range of peptide length in Lu-NEC patients. (B-C) Box plots and ridge plots showing the distribution of protein intensity with log2 transformation in Lu-NECs.


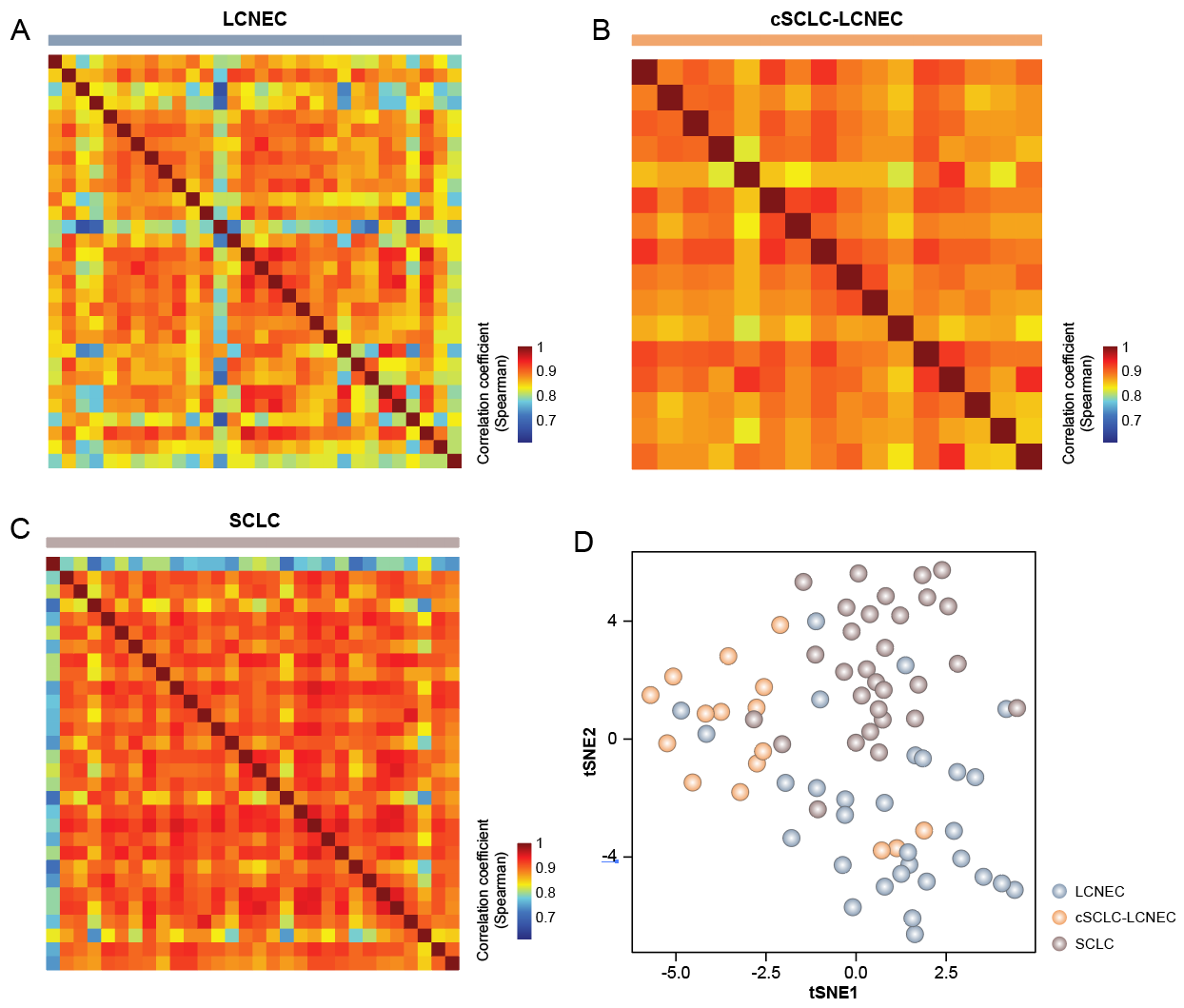


**Supplementary Figure 4. Correlation of proteins in Lu-NECs.** (A-C) Spearman correlation of the intensity of 6,979 proteins between each two samples in Lu-NECs. (D) Two-dimensional t-SNE plot of Lu-NEC samples using the proteomic profile.


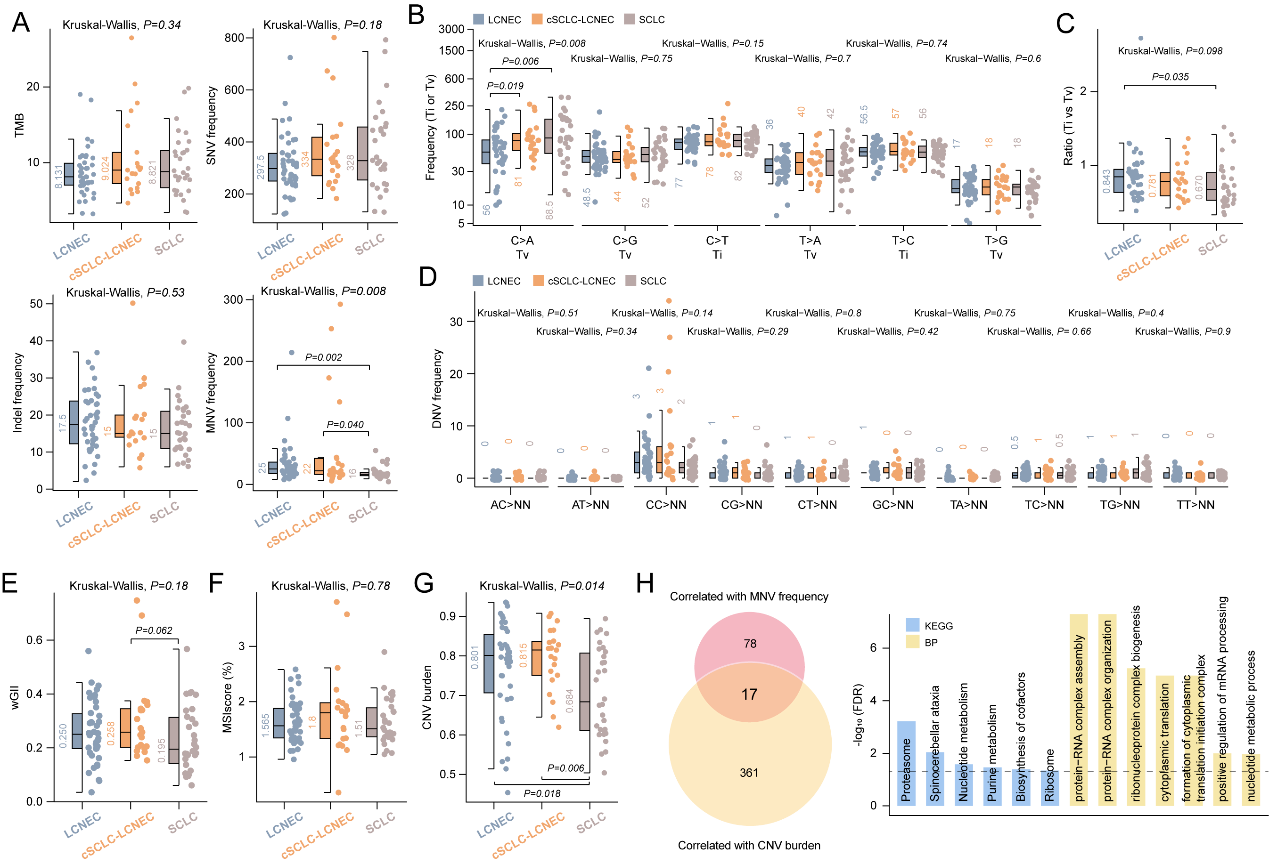


**Supplementary Figure 5. Overview of the mutational landscape between LCNEC, cSCLC-LCNEC and SCLC.** (A) Box plots showing the difference in tumor mutation burden (TMB), single nucleotide variant (SNV) frequencies, indel frequencies and multi-nucleotide variant (MNV) frequencies between LCNEC, cSCLC-LCNEC and SCLC. (B-D) Box plots showing the difference in Ti (or Tv) frequencies, Tv/Ti ratio and DNV frequencies among Lu-NECs. (E-G) Box plots showing the different distribution of weighted genomic instability index (wGII), microsatellite instability score (MSI score %) and copy number variation burden (CNV burden) among three Lu-NEC pathological subtypes. Statistical analyses were performed using the Kruskal-Wallis test for comparisons among all three Lu-NEC subtypes and the Wilcoxon test for comparisons between each two subtypes. (H) The Venn plot showing the number of proteins that were significantly positively correlated with MNV frequency and CNV burden. P-values were calculated using Spearman's rank correlation coefficient method. Bar plots showing the enrichment of biological features including GO BP and KEGG pathway for 17 strong correlated proteins. P-values were adjusted using the FDR method.

**
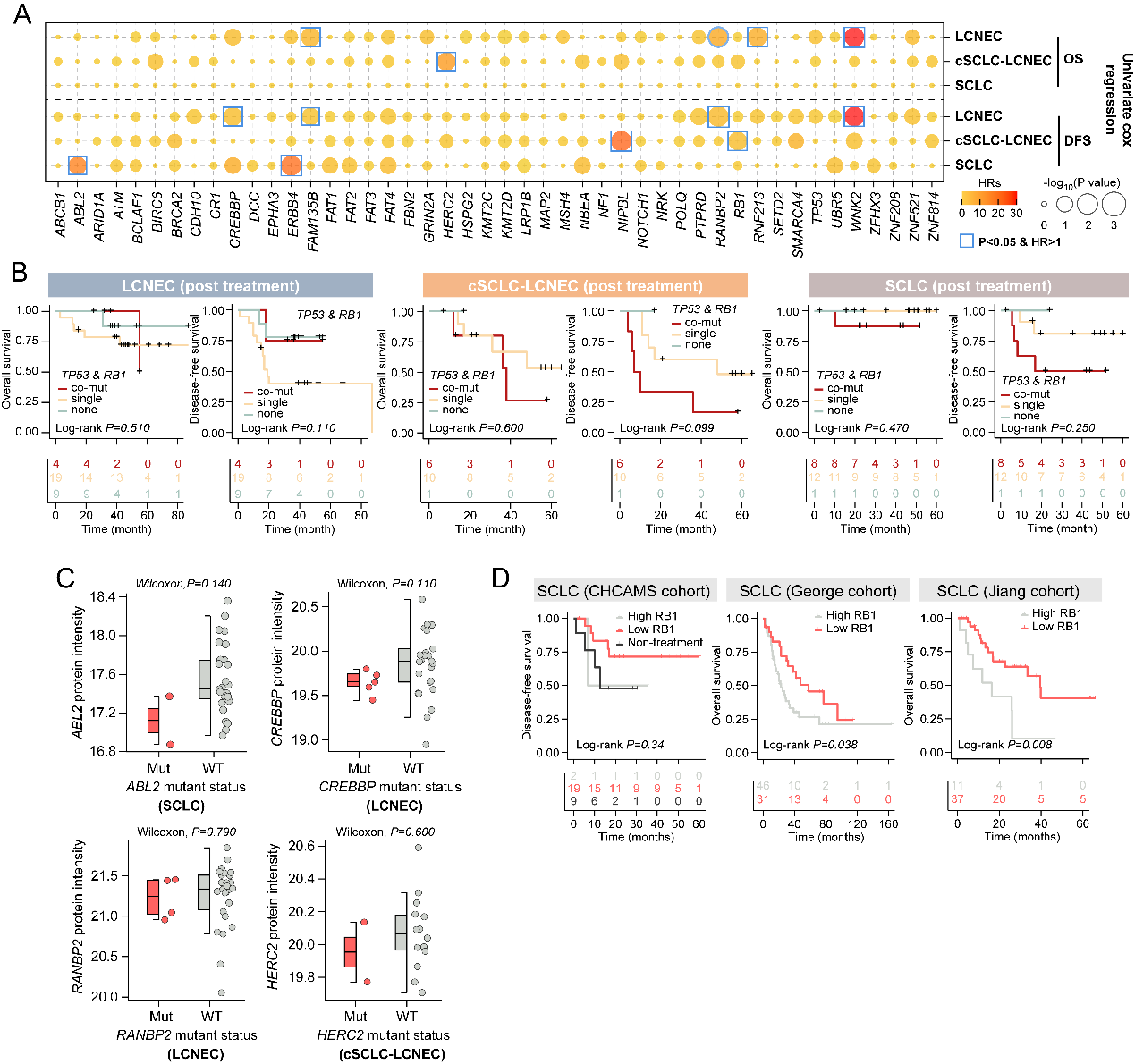
**

**Supplementary Figure 6. Identification of prognostic genomic alterations in Lu-NECs.** (A) Dot plots showing the hazard ratio (HR) and P-value for OS and DFS of tumor driver gene mutation status among Lu-NECs (univariate Cox regression). Color represents HR and dot size represents -log_10_(P-value). The Blue framed line represents significant prognostic tumor driver genes. (B) The Kaplan-Meier curves showing the different prognosis (OS and DFS) of *TP53*&*RB1* mutation status in LCNEC, cSCLC-LCNEC and SCLC (log-rank test). (C) Box plots showing the distribution of tumor driver proteins with cis-effects between corresponding mutation status (Wilcoxon test). (D) The Kaplan-Meier curves showing the different prognosis (OS or DFS) of RB1 expressed group in SCLC (log-rank test).

**
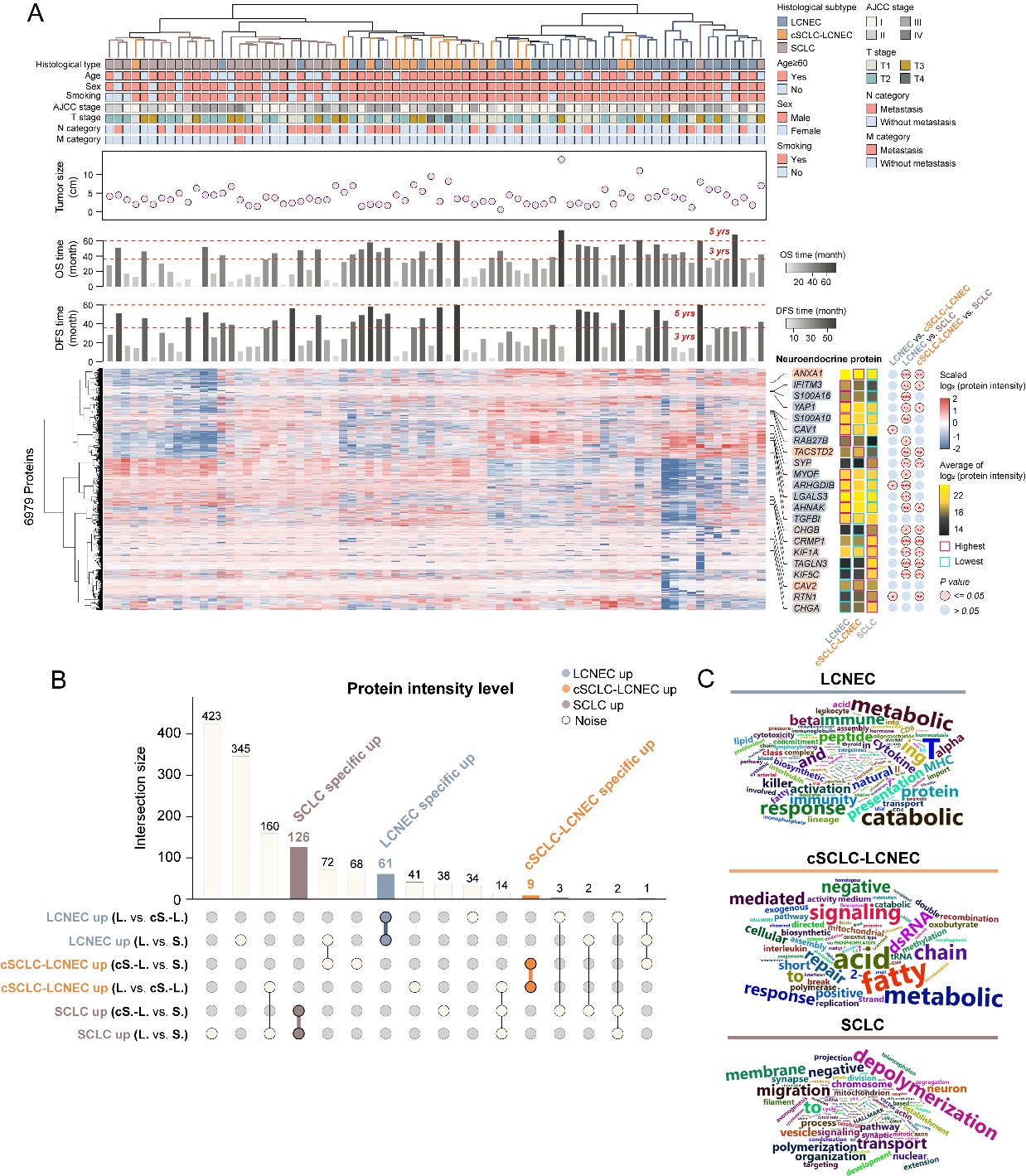
**

**Supplementary Figure 7. Overview of the protein landscape between LCNEC, cSCLC-LCNEC and SCLC.** (A) Heatmap showing the global protein landscape between Lu-NECs and clinicopathological information. (B) Differential expression analysis showing the specific up-regulated proteins in LCNEC (n=61), cSCLC-LCNEC (n=9) and SCLC (n=126). (C) Word cloud analysis showing the primary functional enrichment results in LCNEC, cSCLC-LCNEC and SCLC.

**
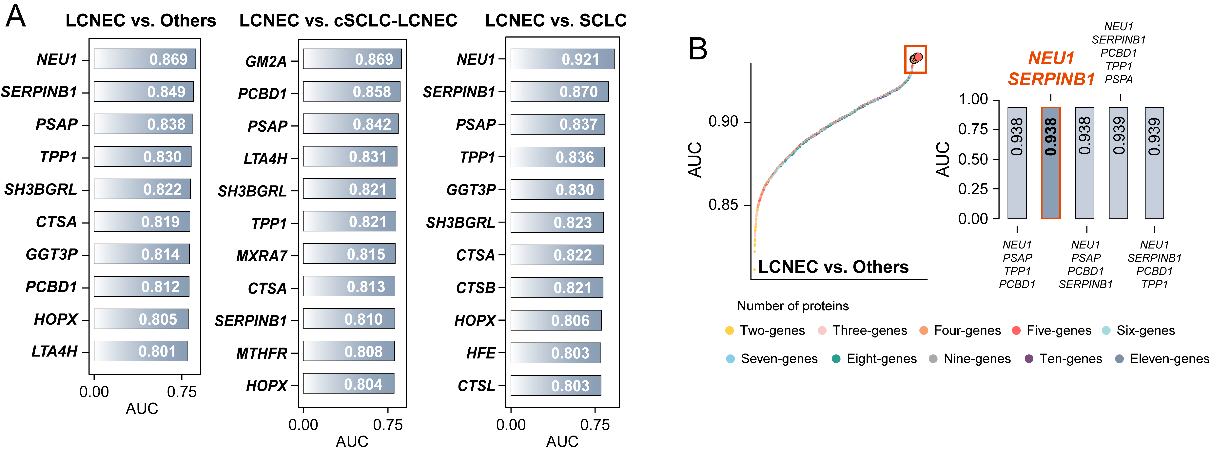
**

**Supplementary Figure 8. Identification of two protein markers.** (A) AUCs of the histological-specific protein marker across Lu-NECs. (B) Comparison for the enumerated combination of the above protein markers in Lu-NECs.

**
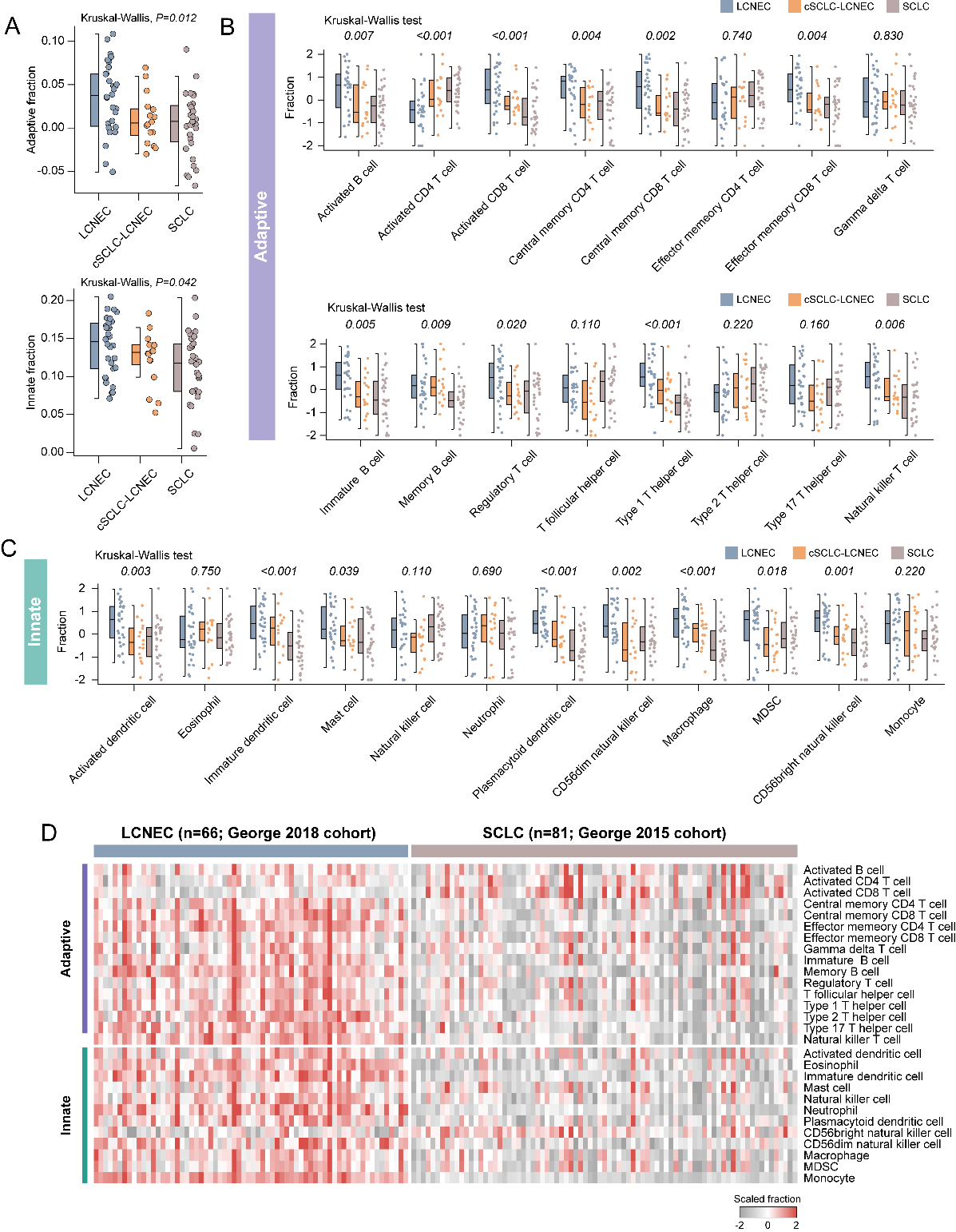
**

**Supplementary Figure 9. Distribution of tumor-infiltrating immune cells in Lu-NECs.** (A) Box plots showing the difference in the adaptive immune fraction and the innate immune fraction between LCNEC, cSCLC-LCNEC and SCLC. (B-C) Box plots showing the distribution of the adaptive immune cell fraction and the innate immune cell fraction in Lu-NECs. Statistical analyses were performed using the Kruskal-Wallis test for comparisons across three pathological subtypes of Lu-NECs. (D) Heatmap showing the distribution of tumor-infiltrating immune cells calculated from LCNEC and SCLC transcriptomic data.


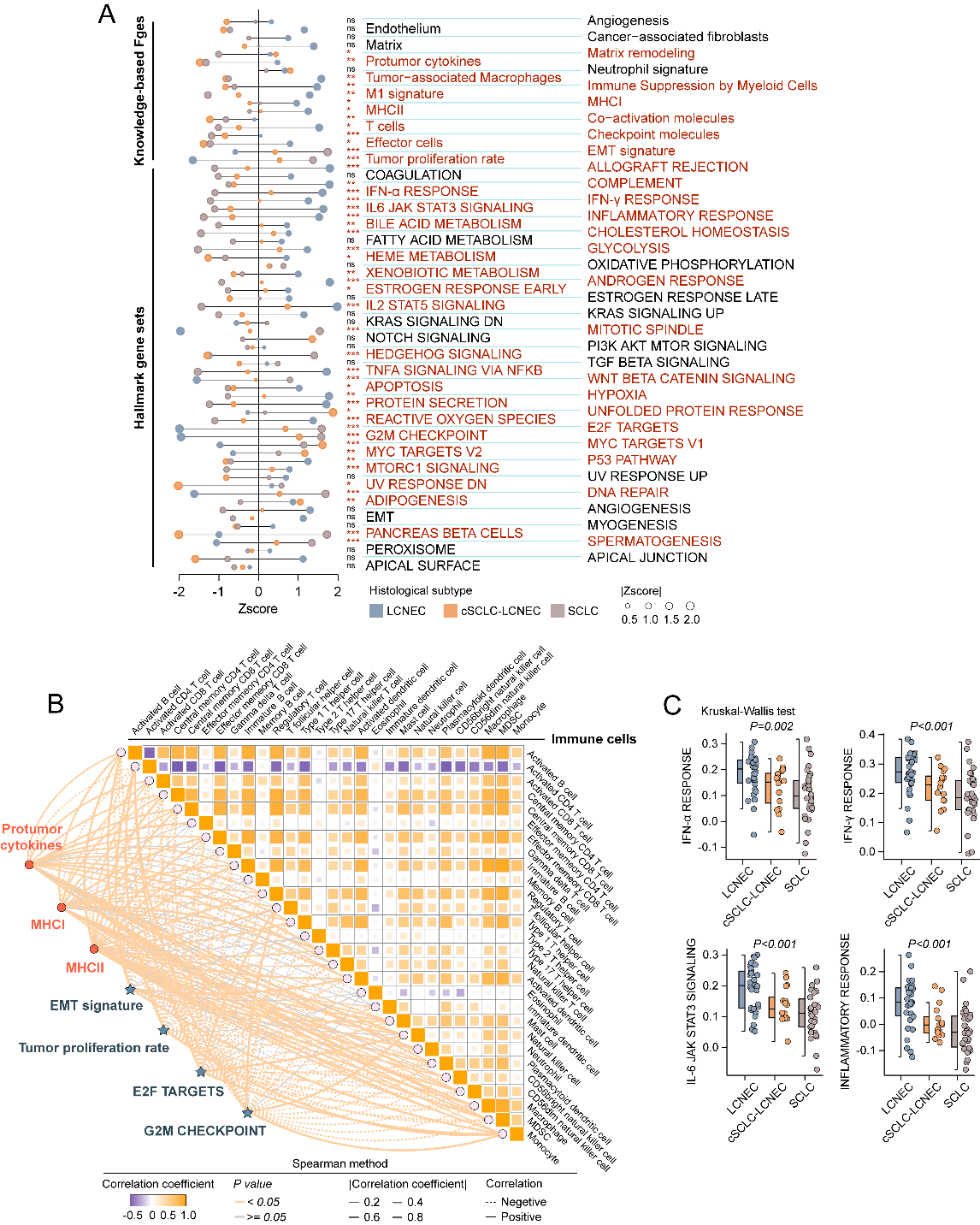


**Supplementary Figure 10.** **Differential tumor microenvironment among three Lu-NEC subtypes.** (A) Dot plots showing the distribution of functional gene set enrichment scores with z-score. A higher score indicates an increase in enrichment in the corresponding Lu-NEC subtype. (B) Heatmap showing the Spearman correlation between immune cell fraction and functional gene set enrichment scores. (C) Box plots showing the differential distribution of IFN-α/IFN-γ response, IL-6 JAK STAT3 signaling and inflammatory response in LCNEC, cSCLC-LCNEC and SCLC (Kruskal-Wallis test).


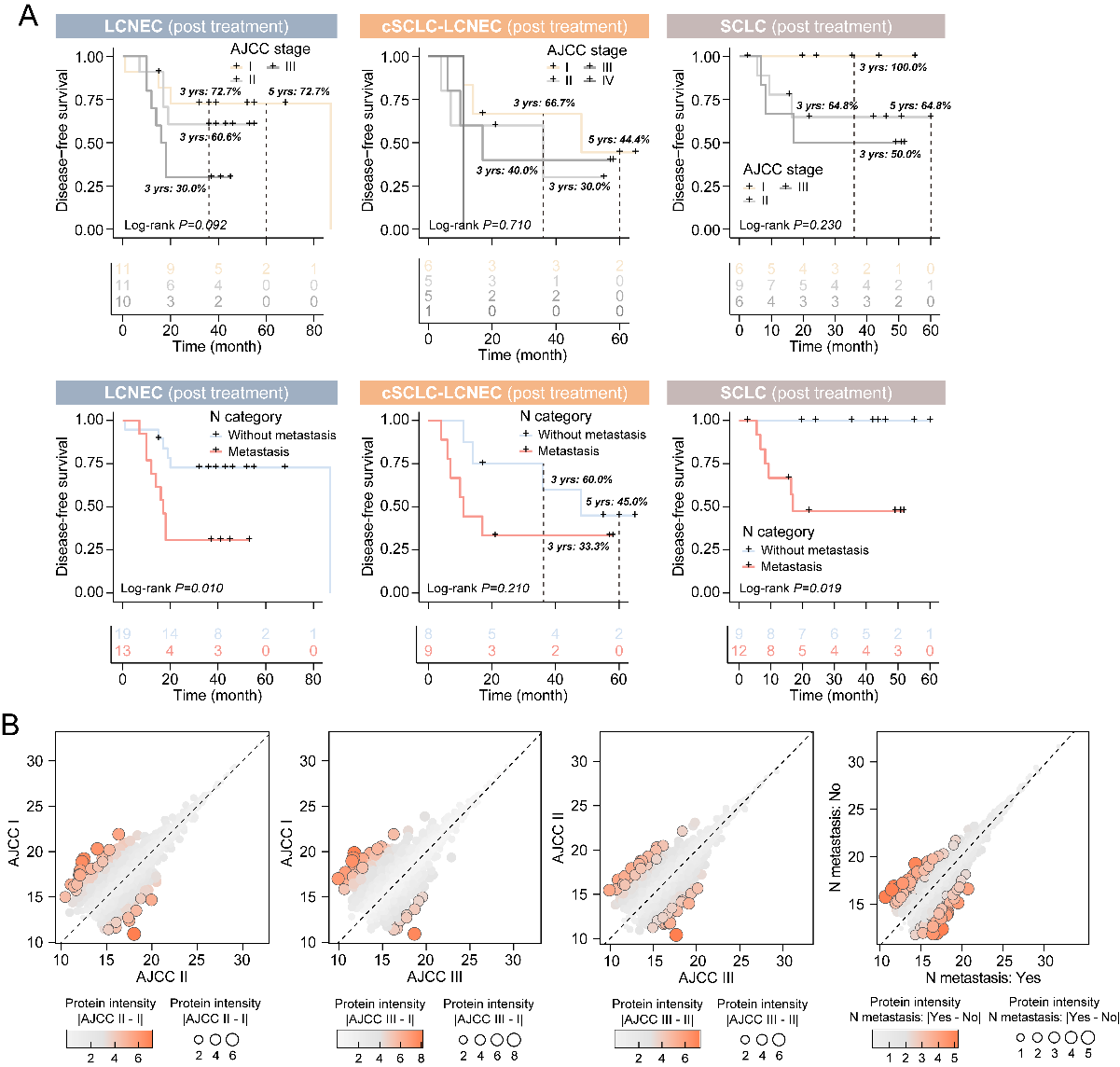


**Supplementary Figure 11. Tumor progression was associated with poor prognosis in LCNEC, cSCLC-LCNEC and SCLC.** (A) The Kaplan-Meier curves showing the different prognoses of the AJCC stage and N category in LCNEC, cSCLC-LCNEC and SCLC (log-rank test). (B) Dot plots showing the difference in protein intensity among AJCC stage and N metastasis. Color and dot size represented the value on the x-axis minus the value on the y-axis.


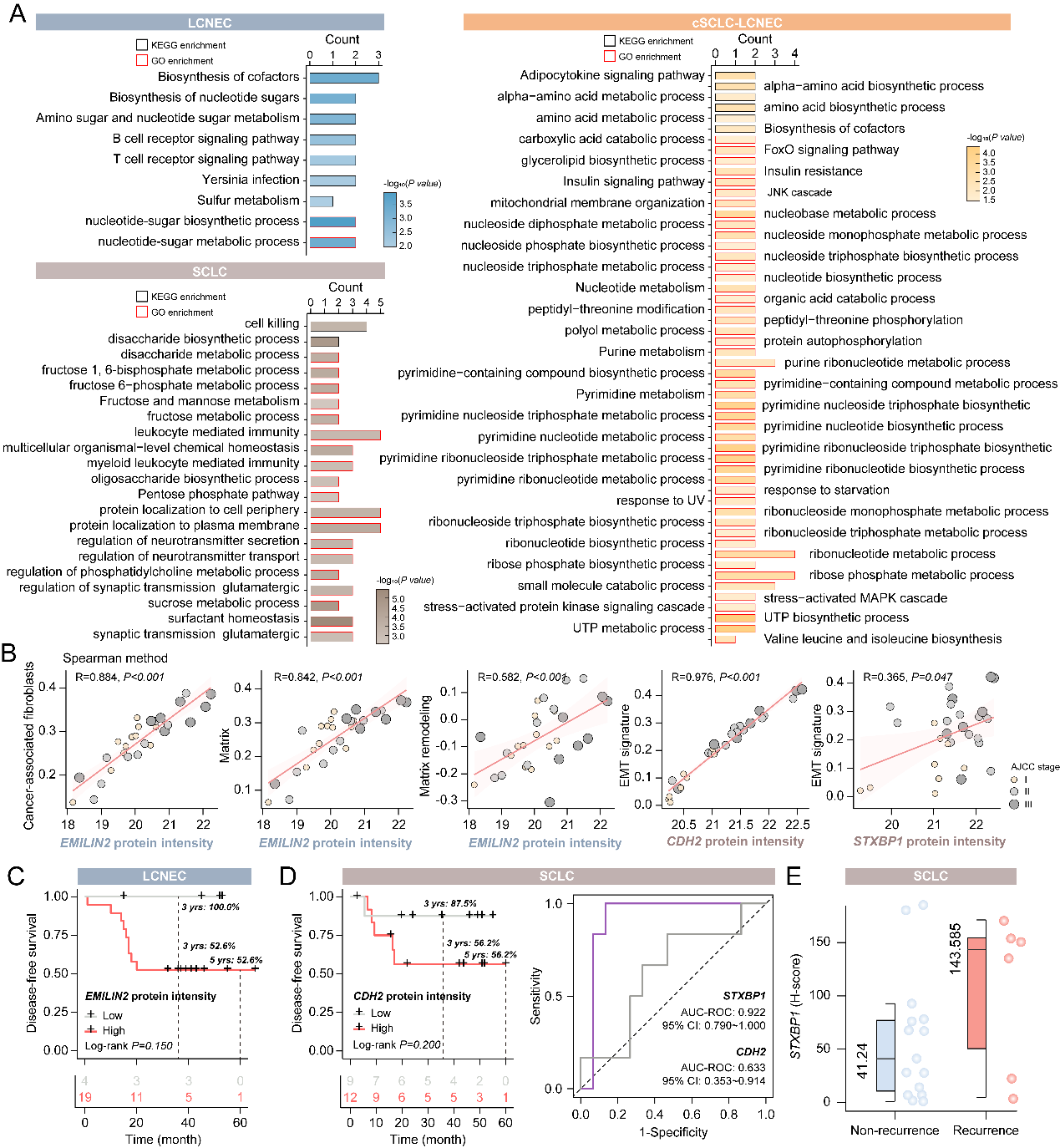


**Supplementary Figure 12. The differential effects of trending proteins among Lu-NEC subtypes.** (A) Bar plots showing the enrichment scores of differentially expressed proteins with monotonic trends in LCNEC, cSCLC-LCNEC and SCLC. (B) Dot plots showing the correlation between progression-associated proteogenomic alterations and oncogenic pathways (Spearman's correlation analysis). (C-D) Kaplan-Meier curves for DFS of proteomic subgroups (log-rank test). ROC analyses showing the predicted performance of recurrence with the protein intensity of STXBP1 and CDH2 in SCLC. (E) Box plot showing the distribution of the H-score of *STXBP1* between recurrence and non-recurrence groups in SCLC.
